# 5‐Hydroxymethylcytosine signature in circulating cell‐free DNA as a potential diagnostic factor for early‐stage colorectal cancer and precancerous adenoma

**DOI:** 10.1002/1878-0261.12833

**Published:** 2020-11-14

**Authors:** ZeWen Xiao, Wendy Wu, Chunlong Wu, Man Li, Fuming Sun, Lu Zheng, Gaojing Liu, Xiaoling Li, Zhiyuan Yun, Jiebing Tang, Yang Yu, Shengnan Luo, Wenji Sun, Xiaohong Feng, Qian Cheng, Xue Tao, Shuangxiu Wu, Ji Tao

**Affiliations:** ^1^ Department of Gastroenterology Harbin Medical University Cancer Hospital China; ^2^ Berry Oncology Corporation Fuzhou China; ^3^ Department of Endoscopic Room Harbin Medical University Cancer Hospital China; ^4^ Department of VIP Ward Harbin Medical University Cancer Hospital China; ^5^ Department of Hematology Harbin Medical University Cancer Hospital China

**Keywords:** 5‐hydroxymethylcytosine signature, cell‐free DNA, early‐stage colorectal cancer, precancerous adenoma

## Abstract

Approximately 85% colorectal cancers (CRCs) are thought to evolve through the adenoma‐to‐carcinoma sequence associated with specific molecular alterations, including the 5‐hydroxymethylcytosine (5hmC) signature in circulating cell‐free DNA (cfDNA). To explore colorectal disease progression and evaluate the use of cfDNA as a potential diagnostic factor for CRC screening, here, we performed genome‐wide 5hmC profiling in plasma cfDNA and tissue genomic DNA (gDNA) acquired from 101 samples (63 plasma and 38 tissues), collected from 21 early‐stage CRC patients, 21 AD patients, and 21 healthy controls (HC). The gDNA and cfDNA 5hmC signatures identified in gene bodies and promoter regions in CRC and AD groups were compared with those in HC group. All the differential 5hmC‐modified regions (DhMRs) were gathered into four clusters: Disease‐enriched, AD‐enriched, Disease‐lost, and AD‐lost, with no overlap. AD‐related clusters, AD‐enriched and AD‐lost, displayed the unique 5hmC signals in AD patients. Disease‐enriched and Disease‐lost clusters indicated the general 5hmC changes when colorectal lesions occurred. Cancer patients with a confirmable adenoma history segmentally gathered in AD‐enriched clusters. KEGG functional enrichment and GO analyses determined distinct differential 5hmC‐modified profiles in cfDNA of HC individuals, AD, and CRC patients. All patients had comprehensive 5hmC signatures where Disease‐enriched and Disease‐lost DhMR clusters demonstrated similar epigenetic modifications, while AD‐enriched and AD‐lost DhMR clusters indicated complicated subpopulations in adenoma. Analysis of CRC patients with adenoma history showed exclusive 5hmC‐gain characteristics, consistent with the ‘parallel’ evolution hypothesis in adenoma, either developed through the adenoma‐to‐carcinoma sequence or not. These findings deepen our understanding of colorectal disease and suggest that the 5hmC modifications of different pathological subtypes (cancer patients with or without adenoma history) could be used to screen early‐stage CRC and assess adenoma malignancy with large‐scale follow‐up studies in the future.

## Graphical abstract

Genome‐wide 5hmC profiling in cell‐free DNA acquired from CRC patients, adenoma patients, and healthy individuals revealed that the differential 5hmC‐modified regions were gathered into four clusters with no overlap, although there are a few overlapped genes shared between the different clusters. CRC patients with adenoma history showed exclusive 5hmC‐gain characteristics, which was consistent with the ‘parallel’ evolution hypothesis in adenoma.

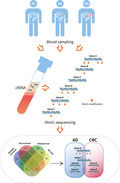

**Abbreviations**
5hmC: 5‐hydroxymethylcytosine
AD: precancerous adenoma
cfDNA: cell‐free DNA
CRC: colorectal cancer
DhmR: differential 5hmC‐modified regions
gDNA: genomics DNA
HC: healthy control
hMRs: 5hmC‐modified regions

## 1 Introduction

Although the overall death rate of colorectal cancer (CRC) continues to drop in developed countries and regions with higher socioeconomic status, it is still the second leading cause of mortality (9.2%) worldwide [1, 2]. Approximately 85% of CRCs are thought to evolve from adenomas through the adenoma‐to‐carcinoma sequence associated with molecular alterations, including hypermethylation. Conversely, only ~ 0.2–0.6% of these premalignant lesions, adenomas, are estimated to transform into adenocarcinoma [3, 4]. About 3.4–7.6% of adenomas have advanced histology (tubular adenoma ≥ 10 MM, adenoma with at least 25% villous feature, high‐grade dysplasia, or carcinoma), with three times higher malignant rate than adenomatous polyps [5]. New evidence is required to further understand the comprehensive biology of colorectal adenoma and cancer, to identify potential targets for tumor screening, and to evaluate persons with average risk for precancerous adenomas.

Circulating cell‐free DNA (cfDNA) in plasma provides a noninvasive diagnostic technique to mirror the genomic information contained in malignant tissue and parallel dynamic changes with different therapies [6, 7], which offers the promise of exquisite sensitivity and specificity for the diagnosis, prognosis, and treatment of cancer [8, 9, 10, 11]. The DNA modification of 5‐hydroxymethylcytosine (5hmC) is a significant DNA epigenetic alteration that affects global gene expression and is involved in tumors, neurodegenerative diseases, and atherosclerosis [12, 13, 14]. Genome‐wide 5hmC distributions and dynamics in various human tissues have shown that it is mainly enriched in the gene body region and has a potential role in gene regulation in mammalian development and cell differentiation [15]. 5hmC was recently determined to be a potential cfDNA biomarker for non‐small‐cell lung cancer and esophageal cancer [16, 17]. In addition, Li *et al*. [18] demonstrated that 5hmC characteristics from plasma cfDNA could distinguish CRC patients from healthy individuals with 83% sensitivity and 94% specificity. Moreover, the robustness of cancer‐specific epigenetic 5hmC signals was identified for colon cancers using different technicians and independent batches of reagents [19]. The present study aims to deeply understand the comprehensive biology of colorectal adenomas and tumors through 5hmC sequencing methodology and to discuss the possibility of using cfDNA 5hmC as candidate biomarkers for the peripheral blood screening and surveillance approach in colorectal adenoma and cancer clinical treatment.

Here, we describe genome‐wide 5‐hmC profiling in cfDNA and gDNA acquired from 101 samples (63 plasma and 38 paired tissue), collected from 21 early‐stage CRC patients (at least seven with adenoma history), 21 precancerous adenoma (AD) patients (17 with advanced histological features), and 21 healthy controls (HCs). A comprehensive review of epigenetic changes in colon disease, adenoma, and early‐stage CRC was conducted. All the differential 5hmC‐modified regions (DhMR) were gathered into four clusters: Disease‐enriched, AD‐enriched, Disease‐lost, and AD‐lost, with no overlap, suggesting that CRC and adenoma displayed different 5hmC signatures. Similar 5hmC modification signals were found in colon patients with differential pathological status (shown in Disease‐enriched and Disease‐lost clusters), and a more complicated 5hmC characteristic was detected in precancerous adenoma, which displayed an elaborate subpopulation in AD (shown in AD‐enriched and AD‐lost clusters). DhMR results of CRC patients with confirmable adenoma history showed exclusive 5hmC‐gain characteristics in the AD‐enriched cluster, which suggested that colorectal adenoma involved a ‘parallel’ evolution rather than a ‘stepwise’ evolution in the adenoma process. This indicates that the tumor screening methodology, using circulating 5hmC cfDNAs as diagnostic biomarkers, should consider various pathological subtypes (i.e., CRC with or without adenoma history) in the future large‐scale follow‐up studies.

## 2 Materials and methods

### 2.1 Study design and sample preparation

A total of 21 CRC patients and 21 precancerous AD patients were diagnosed at the Tumor Hospital at Harbin Medical University, China, from March to September 2018. All tissue specimens were collected from patients who were newly diagnosed and had not received preoperative neoadjuvant therapy, when undergoing surgical removal of tumor tissue or biopsy of precancerous adenoma tissue for pathological diagnosis (Table 1). Paired peripheral blood samples were collected just before surgery. Each methodology performed on human samples was conformed to the standards set by the Declaration of Helsinki. All early‐stage CRC samples were selected for this study, including 5 stage I and 16 stage II, as well as conventional types of adenoma samples including 4 non‐advanced tubular adenoma, 17 advanced tubular adenoma, and villus tubular adenoma (Fig. S1). Both tissue and plasma samples from each CRC and AD patient were tested for sequencing quality, and 1 CRC and 3 AD tissue specimens were filtered out by quality control after sequencing. In addition, 21 peripheral blood samples were collected from HC individuals who visited the clinic for routine physical examination. Written informed consent was obtained from all participants, and the study was approved by the Ethical Committee of Medical Research, Tumor Hospital at Harbin Medical University.

**Table 1:** Clinical characteristics of colorectal cancer (A) and precancerous adenoma patients (B) and healthy individuals (C).

A		
Characteristics	Detail in characteristics	Total (*N* = 21) no. of patients (%)
Age	Median [range]	60 [27–76]
Gender	Male	17 (80.95)
	Female	4 (19.05)
Tumor size	≥ Median (4.5)	12 (57.14)
	< Median (4.5)	9 (42.86)
Stage	I	5 (23.81)
	II	16 (76.19)
CEA	≥ Median (3.11)	9 (42.86)
	< Median (3.11)	8 (38.10)
Adenoma history	Yes	7 (33.33)
	No	14 (66.67)

B		
Characteristics	Detail in characteristics	Total (*N* = 21) no. of patients (%)
Age	Median [range]	53 [39–69]
Gender	Male	14 (66.67)
	Female	5 (23.81)
Tumor size	≥ Median (1.5)	14 (66.67)
	< Median (1.5)	7 (33.33)
Grade	Non‐advanced	4 (19.05)
	Advanced	17 (80.95)

C		
Characteristics	Detail in characteristics	Total (*N* = 21) no. of individuals (%)
Age	Median [range]	55 [40–71]
Gender	Male	17 (80.95)
	Female	4 (19.05)

The tumor tissue and precancerous adenoma tissue were stored at −80 °C after surgical removal. Peripheral blood samples were stored in cell‐free tubes (Streck, Omaha, NE, USA) at 4 °C for no more than 72 h before being separated into plasma and stored at −80 °C. The plasma cfDNA was isolated using the MagMAX Cell‐Free DNA Isolation Kit (Thermo Fisher Scientific, Waltham, MA, USA), and the tissue genomic DNA (gDNA) was isolated using the ZR Genomic DNA Tissue Kits (Zymo Research, Irvine, CA, USA) according to the manufacturer's instructions. The quality of purified DNA was assessed using a Qubit^®^ 4.0 Fluorometer (Life Technologies, Carlsbad, CA, USA), and the DNA fragment size composition was analyzed using a Fragment Analyzer (Agilent, Santa Clara, CA, USA).

### 2.2 5hmC library construction and sequencing

The purified cfDNA (5–20 ng) and gDNA (500 ng) were used to construct a prelibrary. In brief, gDNA was fragmented into DNA fragments of approximately 300 bp using an enzymatic method (5X WGS Fragmentation Mix (Qiagen, Beverly, MA, USA). The fragmented gDNA and cfDNA were end‐repaired and A‐tailed then ligated with T‐adaptors on both ends using 5X ER/A‐Tailing Enzyme Mix and WGS Ligase (Enzymatics), to produce a prelibrary. Then, the 5hmC library was constructed according to a modified method previously described [18]. Briefly, T4 bacteriophage β‐glucosyltransferase was applied to transfer an engineered glucose moiety containing an azide group (N3‐UDP‐Glc) onto the hydroxyl group of 5hmC on DNA fragments. Then, modified 5hmC‐containing DNA fragments were labeled by chemical modification with biotin on the azide group using DBCO‐PEG4‐Biotin (Click Chemistry Tools, Scottsdale, AZ, USA) for further affinity enrichment. PCR amplification was utilized to amplify the captured DNA fragments using M270 beads (Thermo), followed by purification of the PCR products using AMPure XP beads according to the manufacturer's instructions. Finally, sequencing was performed using the Illumina NovaSeq 6000 platform (Illumina, San Diego, CA, USA).

In this study, two similar spike‐in probes with unique sequences, named 5hmC spike‐in and no5hmC spike‐in, were designed and used for library construction and sequencing, to calculate 5hmC‐DNA capture and enrichment efficiency.

Note: 5hmC spike‐in sequence: 5′‐CGACCGAGTTGCTCTTGCCC*GGCGTCAACACGGGATAATACCGCGCCACATAGCAGAACTTTAAAAGTGCTCATCATTGGAAAACGTTCTTCGGGGCGAAAACTCTCAAGGATCTTACCGCTGTTGAGAT‐3′; C* means 5hmC modifications. no5hmC spike‐in sequence: 5′‐CCTGAAGTCCGGCTGGAGTGAGTGGGAAGAGAGCGCCACGGACAGTATGTCGCAGGTAAAAAGTGCAGCCACGCAGACCTTTGATGGTATTGCACAGAATATGGCGGCGATGCTGACCGGCAGTGAGCAG‐3′. These sequences could not be mapped to the human reference genome. The 5hmC and no5hmC spike‐in sequence DNA fragments were mixed with the experimental sample before library preparation. After sequencing, the spike‐in reads were extracted and the enrichment ratios for each sample were calculated.

### 2.3 Sequencing data processing

The raw sequencing reads were removed with low‐quality (less than Q20) reads and aligned to the human reference genome (hg19/GRCh37). Model‐based analysis of ChIP‐seq [20] was used to identify the 5hmC‐enriched regions in each sample (the *q* value cutoff for significant regions was 0.01; model fold = [5, 50]). The sample with 5hmC‐DNA capture and enrichment efficiency higher than 100 folds and mapping rate of more than 90% was used for further analysis. Peaks with high enrichment and significance (*q* < 1E‐12; fold enrichment > 8) in all samples were considered as highly reliable peaks and were combined into one unified catalog by the ‘mergePeak’ function from homer (version 4.9.1) [21] (merged peaks: 3095 for CRC plasma samples, 2241 for HC plasma samples, 2737 for AD plasma samples, 225 for CRC tissue samples, and 261 for AD tissue samples).

The genome was divided into 2 kb windows, and 5hmC enrichment levels in each window were expressed as fragments per kb of 5hmC‐DNA per million fragments mapped (FPKM). The genomic annotation of 5hmC peak regions was performed using annotatr [22]. The metagene profile of the median 5hmC peak level was generated using ngsplot [23].

### 2.4 Differential 5hmC peak region detection and functional annotation

Genes with differential 5hmC levels were detected in all the tissue and plasma samples using the software edger of the r package [24]. The density distribution of 5hmC peak number was calculated by the distribution of the observational frequencies of all peaks. The differential 5hmC peak regions between the HC, AD, and CRC groups were identified with fold change (FC) > 1.5 and *P* value < 0.05. A heatmap of r package (https://cran.r‐project.org/web/packages/pheatmap/index.html) was used to visualize hierarchical clustering and the distance in a heatmap figure [25]. Gene Ontology (GO) term analyses were performed using r package ‘topgo’ (http://www.bioconductor.org/packages/release/bioc/vignettes/topGO/inst/doc/topGO.pdf), and KEGG pathway enrichment was conducted using kobas online tools (http://kobas.cbi.pku.edu.cn/) [25].

## 3 Results

### 3.1 5hmC modification profiling of clinical specimens

**Fig. 1:**
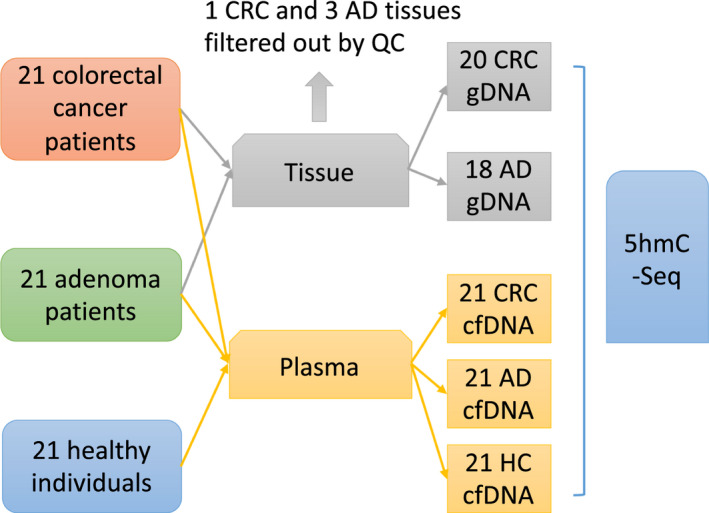
Schematic flowchart of sample collection and filtering.

We first compared the 5hmC features of cfDNA in plasma among early‐stage CRC, AD, and HC groups, and gDNA in tissue of the CRC and AD groups using the sensitive 5hmC sequencing method (Fig. S2) [26]. The protocol comprised of DNA extraction, adapter ligation, selective labeling, click chemistry, 5hmC capture, and PCR amplification. The 5hmC profiles of cfDNA and gDNA were acquired from 63 plasma and 38 tissue samples, respectively, collected from 21 CRC patients (at least seven with adenoma history), 21 AD patients (17 with advanced histological features), and 21 HC individuals (Fig. 1). Detailed information regarding subject characteristics, tumor features, and adenoma characteristics is illustrated in Table 1 and Table S1. The age and gender distributions among the three groups were unbiased based on the Kruskal–Wallis *H*‐test (Fig. S3). Both tissue and plasma samples from each CRC or AD patient were tested for sequencing quality, and one CRC and three AD tissue specimens were filtered out by quality control after sequencing.

### 3.2 Genome‐wide distribution of 5hmC modifications

**Fig. 2:**
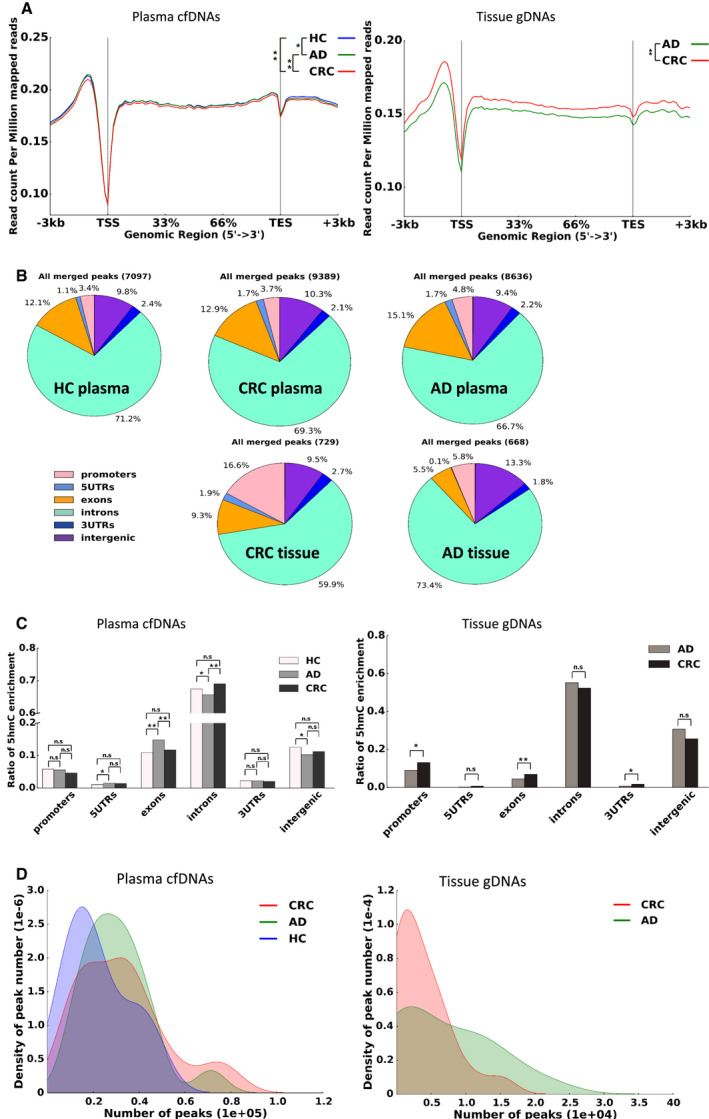
Genome‐wide distribution of 5hmC modifications in cfDNA and gDNA (A) Metagene profiles of mean values of 5hmC read count in all plasma and tissue samples. The left is the cfDNA metagene profile in CRC, AD, and HC plasma. The right is the gDNA metagene profile in CRC and AD tissue. Wilcoxon signed rank test was used to determine significance. (B) The overall 5hmC peak distribution of hMRs. From left to right on the top: plasma from HC individuals, CRC patients, and AD patients. From left to right on the bottom, tumor tissue and adenoma tissue. (C) The column diagram of the 5hmC enrichment score across distinct genomic regions. Mann–Whitney rank test was used to determine significance. The differences of CRC vs AD gDNA in the promoter, exon, and 3′ UTR regions are 0.02, 0.01, and 0.01, respectively. The differences of CRC vs AD cfDNA in the exon and intron regions are 0.001 and 0.002, respectively. The differences of CRC vs HC cfDNA in the 5′UTR, exon, intron, and intergenic regions are 0.03, 4.66E‐05, 0.02, and 0.03, respectively. All other differences are not significant. (D) Density distribution of the peak number. The left from cfDNA and the right from gDNA in (C) and (D), respectively.

High‐throughput sequencing revealed that the global cell‐free 5hmC features in plasma were different among early‐stage CRC, AD, and HC groups. The genome‐wide distribution of 5hmC metagene profiles of cfDNAs (left in Fig. 2A) revealed that 5hmC modification levels in CRC patients were lower than those in HC and AD patients with *P* values of 2.75E‐18 and 2.67E‐18 in Wilcoxon signed rank. The 5hmC signal difference between the HC and AD groups was less significant, with a *P* value of 0.01. Figure 2B shows the results of 5hmC‐modified regions (hMRs) analysis carried out in cfDNAs for the CRC, AD, and HC groups, as well as gDNAs for the CRC and AD groups. We identified 9389, 8636, and 7097 hMRs in plasma from CRC patients, AD patients, and HC individuals, respectively. The majority of these hMRs were located in the intragenic and promoter regions, whereas fewer were found in the intergenic regions, which is consistent with previous investigations [17, 27]. In the tissue samples, 729 and 668 hMRs were identified from the CRC and AD groups, respectively, and aligned mostly to the transcript unit regions. We observed that more hMRs were enriched in the promoter and exon regions of CRC tissue (16.6% and 9.3%) than those of AD tissue (5.8% and 5.5%), but there was not much difference among plasma samples of the CRC, AD, and HC groups. The 5hmC‐modified regions enriched in the promoter and exon regions were more in tissue than plasma, which could attribute to that cfDNA was depleted more at transcription start sites and transcription factor binding peaks compared to the flanking areas.

To further analyze this highly gene‐body‐enriched feature of 5hmC in cfDNA and gDNA, we enumerated the 5hmC enrichment ratio across differential genomics regions (i.e., for each group, 5hmC‐enriched peaks in each gene body region of each sample compared to those of all gene body regions of that sample) (Fig. 2C). The 5hmC peaks in cfDNA and gDNA were both mainly distributed in the intron regions, followed by intergenic regions or exon regions. However, the 5hmC signals from gDNA of early‐stage CRC patients were higher in the promoter and exon regions than those of AD patients (*P* value of 0.02 and 0.01, respectively, based on the Mann–Whitney rank test, right in Fig. 2C), which is inconsistent with the cfDNA results of the CRC and AD groups (*P* value of 0.14 and 0.001, respectively, left in Fig. 2C). We also calculated the density distribution of 5hmC peak number in five sample groups, and Fig. 2D demonstrates the diverse distribution of 5hmC peak density among the three plasma samples with a median number of 33 197, 28 881, and 16 770 for the CRC, AD, and HC groups, respectively. The CRC group exhibited the broadest distribution, while the HC group displayed the sharpest. In contrast, the density of the 5hmC peak number in CRC tissue showed a narrower and sharper curve compared with the AD hMR result.

### 3.3 Pathogenic‐associated 5hmC characteristics in cfDNA among the CRC, AD, and HC groups

**Fig. 3:**
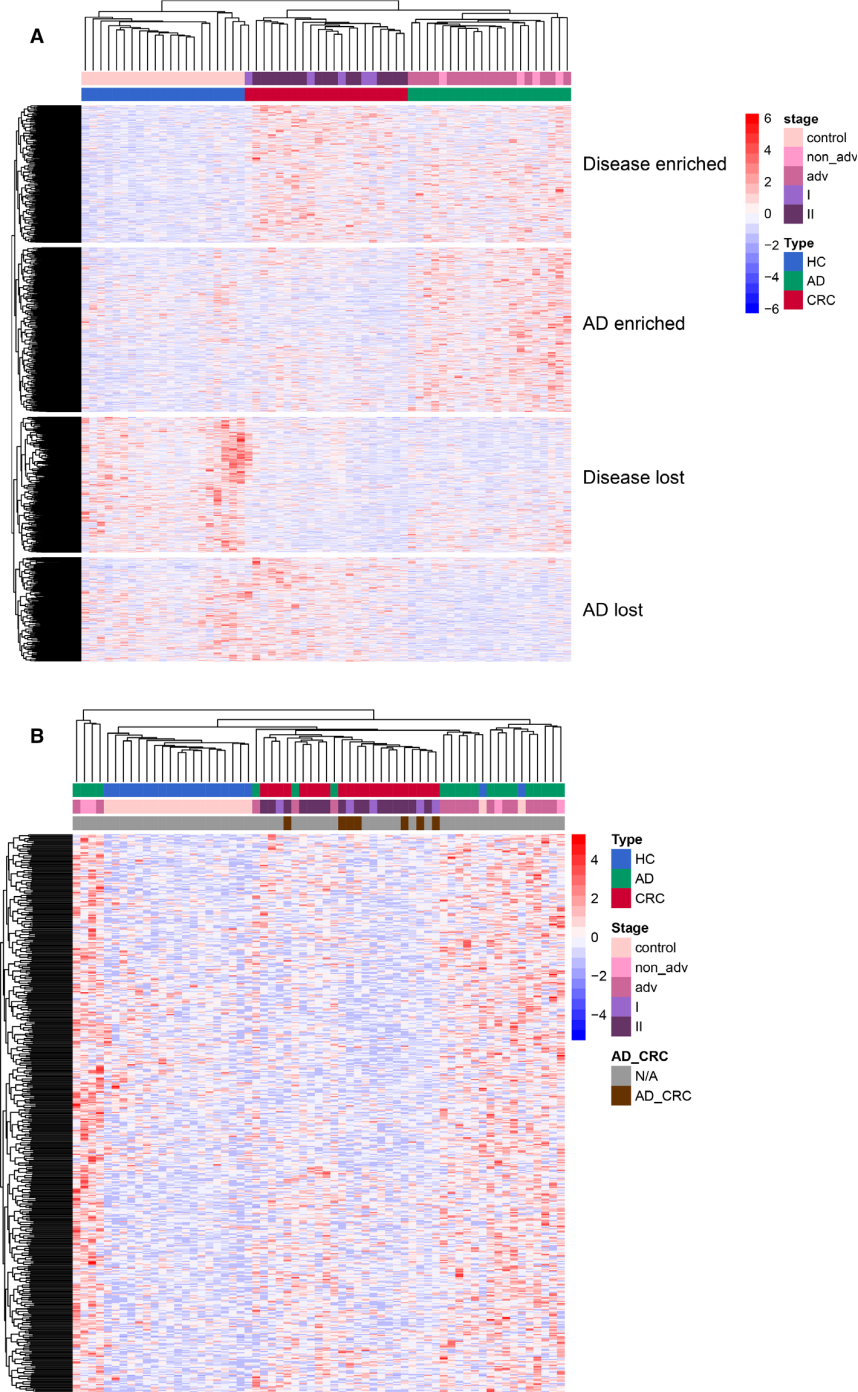
Unsupervised hierarchical clustering was performed across samples and hMRs [fold change (FC) ≥ 2 and *P* ≤ 0.01] with stage status (Stage I and II in CRC; non‐advanced and advanced AD). (A) Heatmap of DhMRs in CRC, AD, and HC groups. (B) Heatmap in AD‐enriched cluster.

To compare the cfDNA 5hmC modification changes among the CRC, AD, and HC samples, we identified the DhMRs across these three groups with the thresholds of FC ≥ 2 and *P* value ≤ 0.01 (Fig. 3A). Unsurprised hierarchical clustering was performed across all samples with detailed pathological status (Fig. S1), stage I or II in CRC patients and non‐advanced or advanced AD patients. All cfDNA DhMRs result was visualized in a heatmap figure (Fig. 3A), which distinctly gathered into four clusters, Disease‐enriched, AD‐enriched, Disease‐lost, and AD‐lost. None of the DhMRs overlapped with each other, and each cluster demonstrated its exclusive trend. Disease‐enriched cluster had higher 5hmC tags in CRC and AD patients but lower in the HC group; AD‐enriched cluster showed higher 5hmC signals in AD samples but lower in the CRC and HC groups; Disease‐lost cluster had higher 5hmC signature in the HC group but lower in the CRC and AD groups; and AD‐lost cluster had less 5hmC reads in AD patients than in the CRC and HC groups. Two AD‐related clusters, AD enrich and AD‐lost, displayed the unique 5hmC signals in precancerous adenoma patients. And Disease‐enriched and Disease‐lost clusters indicated the general 5hmC changes when colorectal lesions occurred. There was no difference of DhMRs between the stage I and II CRC patients, as well as between the non‐advanced and advanced AD patient statuses. We further analyzed the DhMRs in each cluster one by one and discovered CRC patients with a confirmed history of adenoma were segmentally gathered in the AD‐enriched cluster (Fig. 3B), while rest of the patients were scattered in the other three clusters (Fig. S4).

**Fig. 4:**
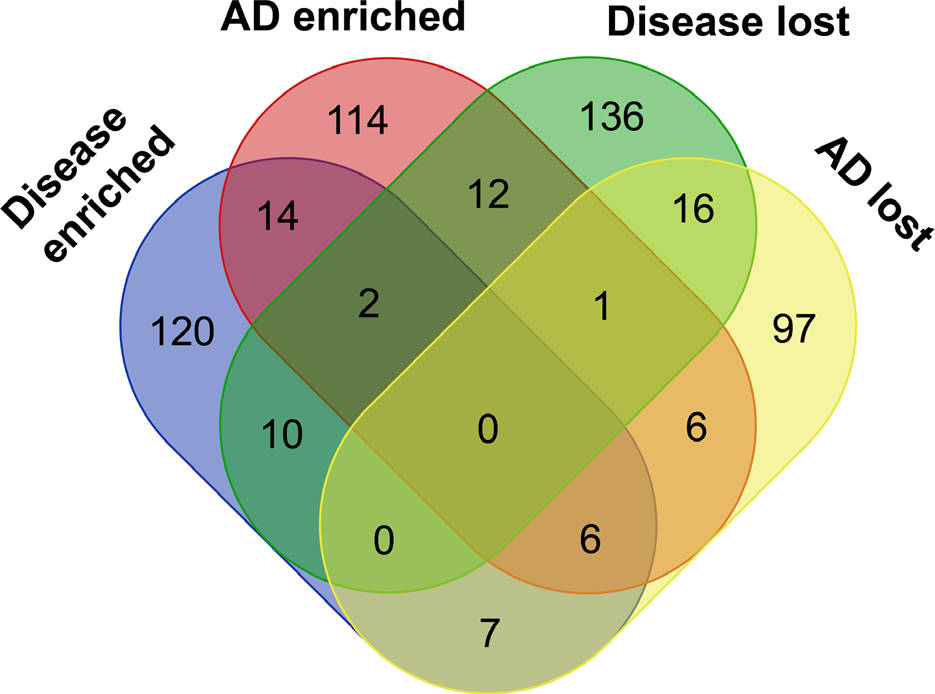
Venn diagram of DhMR‐annotated genes overlap in 4 clusters, Disease‐enriched, AD‐enriched, Disease‐lost, and AD‐lost.

All DhMRs were annotated to gene, and most annotated genes were found to be exclusive in each cluster (Fig. 4), 120 for Disease‐enriched, 114 for AD‐enriched, 136 for Disease‐lost, and 97 for AD‐lost. Shared genes between 2 clusters accounted for a small portion (Table S3), 14 for Disease‐enriched and AD‐enriched (both higher 5hmC tags in AD), 10 for Disease‐enriched and Disease‐lost, 7 for Disease‐enriched and AD‐lost (both higher 5hmC signals in CRC), 12 for AD‐enriched and Disease‐lost (both lower 5hmC signature in CRC), 6 for AD‐enriched and AD‐lost, and 16 for Disease‐lost and AD‐lost (both lower 5hmC feature in AD). Few DhMR‐annotated genes emerged in three clusters (Table S3); 2 for Disease‐enriched, AD‐enriched, and Disease‐lost; 6 for Disease‐enriched, AD‐enriched, and AD‐lost; and 1 for AD‐enriched, Disease‐lost, and AD‐lost. No annotated gene was found in any of the four clusters. The 5hmC characteristics in the early‐stage CRC group were more comprehensive and had two trends: One was similar to the HC (AD‐enriched and AD‐lost), and the other was similar to AD patients (Disease‐enriched and Disease‐lost), implying two different carcinogenesis of CRC based on 5hmC features.

### 3.4 Functional annotation of differential 5hmC change regions in cfDNA among the CRC, AD, and HC groups

**Fig. 5:**
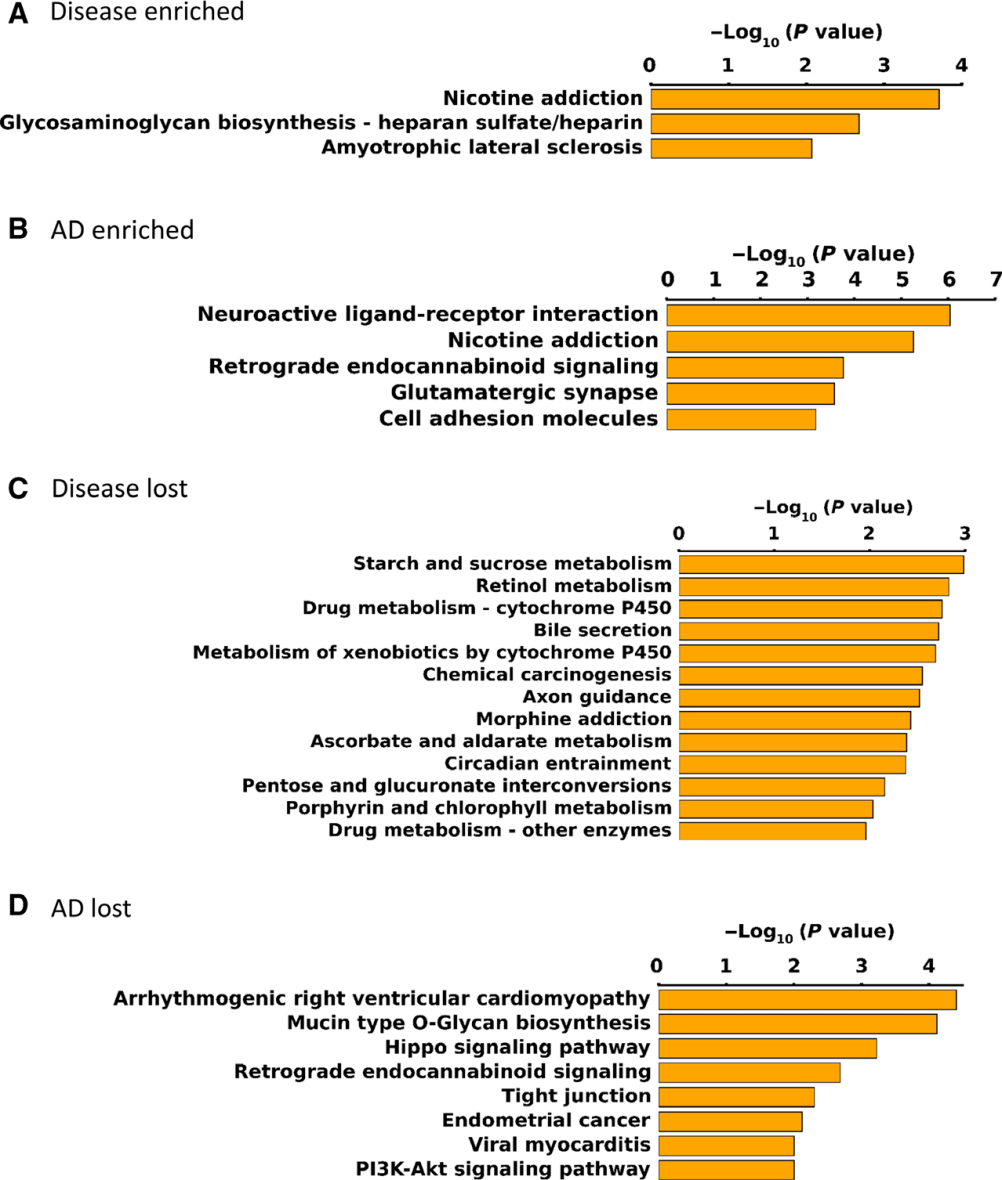
KEGG enrichment analysis (*P* ≤ 0.01) of significant 5hmC‐regulated regions in Disease‐enriched (A), AD‐enriched (B), Disease‐lost (C), and AD‐lost (D).

KEGG pathway enrichment and annotation analysis was performed on four clusters, Disease‐enriched, AD‐enriched, Disease‐lost, and AD‐lost. Figure 5 shows the enriched pathways with a *P* value lower than 0.01. Each cluster had exclusive pathways with two exceptions, retrograde endocannabinoid signaling [28] shown in AD‐enriched and AD‐lost, and nicotine addiction [29, 30] in Disease‐enriched and AD‐enriched. To gain insight into the detailed 5hmC changes in cfDNAs, KEGG functional enrichment analysis in three one‐to‐one comparisons was carried out. Nicotine addiction and retrograde endocannabinoid signaling were both shown in upregulated DhMR‐enriched pathways between CRC patients and HC individuals (Fig. S5A), along with neuroactive ligand–receptor interaction [31, 32], axon guidance [33, 34], and glutamatergic synapses [35], all of which are linked to cancer development. All these pathways were also found in the comparison sets of CRC vs. AD (Fig. S5B) and AD vs. HC (Fig. S5C).

In the reciprocal statistics of DhMR comparison among CRC patients, AD patients, and HCs, that is CRC vs. HC, CRC vs. AD, and AD vs. HC (Fig. S6 and Table S4), all genes in the top 20 DhMRs (Table S5) were identified according to the *P* values. Most genes are associated with cancer pathogenesis. For instance, the top 1 gene in DhMRs between CRC patients and HCs was TP63, a new promising therapeutic member of the well‐known TP53 family, which can suppress tumor cell growth, induce cell apoptosis, and cooperate with chemotherapy with low dose and less side effects [36]. The top hit between AD and HC groups, KLK4, kallikrein‐related peptidase 4, has been implicated in many types of cancer and has potential as a tumor biomarker [37, 38, 39] which also predicts short‐term relapse in colorectal adenocarcinoma patients [40]. These annotated genes of the top 20 DhMRs among the three comparison sets underwent unsupervised hierarchical clustering (Fig. S7) and revealed distinction in comparison.

We also performed GO function annotation analysis on four clusters. The most significant GO function items hit by DhMRs of each cluster fall into the cell periphery, plasma membrane, postsynaptic membrane, cytoplasm, dendrite, axon, cell junction, Golgi apparatus, etc. in cell component, accounting for a broad function of various protein bindings in molecular function as well as cellular signaling transduction, axon guidance, cell adhesion, immune response, regulation of apoptotic process and cell proliferation, skeletal system development, small molecule metabolic process, and protein phosphorylation in biological processes (Table S6).

## 4 Discussion

We sequenced cfDNA to generate genome‐wide 5hmC profiles in the plasma of CRC patients, precancerous AD patients, and HC individuals, and sequenced the gDNA in the corresponding CRC and AD tissues. The robust tumor‐associated gDNA and cfDNA 5hmC signatures were identified in the gene body and promoter regions of the CRC and AD groups compared with the HC group. The majority of genome‐wide hMR distribution in all three plasma samples were observed in the intragenic and promoter regions, whereas fewer were found in the intergenic regions, which is consistent with the results of previous investigations [17, 27]. The 5hmC metagene profiles in CRC gDNAs were higher than those in the AD group in the entire genomic region (*P* value of 2.67E‐18), whereas the cfDNA 5hmC metagene profiles showed an overall reduction in CRC, consistent with previous studies [41, 42]. More CRC tissue hMRs were enriched in promoter (16.6%) and exon (9.3%) regions than in AD tissue hMRs (5.8% and 5.5%). This phenomenon indicated that the 5hmC‐signal changes in gDNAs between CRC and AD are quite remarkable, but might be heavily diluted in the plasma cfDNAs or with different 5hmC modification features in cfDNAs. The diverse distribution of 5hmC peak number density among the three plasma samples illustrated a gradual widening distribution in cfDNA 5hmC enrichment from HC to AD then CRC, and CRC density of 5hmC peak number in tissue showed a narrower and sharper curve compared with the AD result. The irrelevance of the epigenetic features between plasma cfDNA and tissue gDNA for 5hmC signals was also found in stomach and colon cancers reported by Li *et al*. [18]. All the 5hmC DhMR identified in our cohort had been performed on the CRC and HC cohort studied by Li *et al*. [18]. The cancer patients and the healthy individuals were clustered quite clear (data not shown), which could reinforce our results.

All the cfDNA DhMRs were gathered into four clusters: Disease‐enriched, AD‐enriched, Disease‐lost, and AD‐lost, with no overlap. This suggests that the cfDNA 5hmC modifications among HCs, AD patients, and CRC patients are quite distinct. None of the clusters could separate pathologic subtypes of stage I and II CRC or non‐advanced and advanced adenoma, and the comparison result in different stages CRC was similar to a previous investigation of CRC [18]. The KEGG functional enrichment results also indicated that the HC, AD patients, and early‐stage CRC patients mostly had very different DhMR profiles in cfDNA. But nicotine addiction, retrograde endocannabinoid signaling, neuroactive ligand–receptor interaction, axon guidance, and glutamatergic synapses might involve in AD malignance and CRC origin (Fig. S5).

The 5hmC signature in the CRC group was more comprehensive and contained two features: one similar to the HC group (AD‐enriched and AD‐lost), and the other similar to the AD patients (Disease‐enriched and Disease‐lost). This was in agreement with previous evidence that a major part of CRCs evolved through the adenoma‐to‐carcinoma sequence, and the remainder did not [3, 43]. AD patients involved in this study exhibited an independent property in their 5hmC profiles compared to CRC patients and HC individuals, and CRC patients with a confirmable adenoma history segmentally gathered in the AD‐enriched cluster. As we know, adenoma has various pathological subtypes and only about 5% of adenoma with advanced histology (tubular adenoma ≥ 10 MM, adenoma with at least 25% villous features, or high‐grade dysplasia or carcinoma) would be malignant [5]. Conversely, approximately 85% of CRCs are thought to arise from the adenoma‐to‐carcinoma sequence related to molecular alterations, including hypermethylation [3]. It had been suggested that colorectal adenoma involved a ‘parallel’ evolution rather than a ‘stepwise’ evolution in progression [44], which implied that variable clonal subpopulation in adenoma derived from two ways, one being the conventional adenoma‐to‐carcinoma sequence, and the other regressed from the malignant progress [45]. The clonal dynamics could be similar to intratumoral heterogeneity arising through the evolution of genetically diverse subclones during the tumor process [46]. Our work provides a global overview of 5hmC modification changes in early‐stage CRC, AD, and HC individuals and offers an insight on using 5hmC signals of various pathological subtypes, including CRC with or without adenoma history, for early CRC diagnosis and adenoma malignancy evaluation. However, the limitation of the low number in this cohort should be noted. To further investigate the potential application of cfDNA 5hmC signature in clinical prevention, a large‐scale long‐term cohort study must be performed and included more proportion of CRC patients with and without confirmable AD history.

## 5 Conclusions

In summary, we generated 5hmC profiles of cfDNA from Chinese CRC patients, AD patients, and HC individuals, as well as the gDNA 5hmC profiles for tumor and adenoma tissue. Large‐scale 5hmC changes in promoter and gene body regions were detected. All the cfDNA DhMRs were gathered into four clusters: Disease‐enriched, AD‐enriched, Disease‐lost, and AD‐lost, which did not overlap. CRC patients with a confirmable adenoma history segmentally gathered in the AD‐enriched cluster. This provides a comprehensive and global overview of epigenetic changes in colon disease states between adenoma and early‐stage CRC. It suggested that there are two pathologic subtypes of precancerous adenoma: one could progress to malignant lesion through adenoma‐to‐carcinoma sequence and the other not. The 5hmC modification changes with clear pathological evidences, that is, CRC with or without adenoma history, could expand the molecular biological knowledge of the colorectal disease process and benefit early‐stage CRC screening. This finding suggests the potential values of using 5hmC as diagnostic biomarkers to develop new strategies for CRC diagnosis through large‐scale studies on patients with clear colorectal disease history and long‐enough follow‐up surveys to evaluate the possibility of adenoma malignancy.

## Conflict of interest

Shuangxiu Wu, Wendy Wu, Lu Zheng, Gaojing Liu, Xiaoling Li, and Fuming Sun are the employees of BerryOncology Corporation. The other authors declare that they have no conflict of interest.

## Author contributions

JT and ZX conceived and designed the study. JT, SW, and ZX designed the methodology. LZ and XL performed the laboratory experiments. FS, GL, SW, and WW analyzed the data. WW and SW wrote and revised the manuscript. ZX, JT, CW, ML, and ZY involved in acquisition of material (reagents, animals, and clinical samples). JT, YY, SL, WS, XF, QC, and XT gave the important intellectual input. JT supervised the study.

## Supporting information


**Fig. S1.** Representative images of colorectal cancer (Stage I and Stage II), advanced tubular adenoma, Villus tubular adenoma and non‐advanced adenoma tissue.
**Fig. S2.** General procedure of 5hmC sequencing profiling from gDNA and cfDNA.
**Fig. S3.** Kruskal‐Wallis H‐test among CRC, AD and HC groups (A) for age distributions, (B) for gender distributions.
**Fig. S4.** Heatmap of differentially 5hmC hMRs (FC *≥* 2 and p ≤ 0.01) with stage status (Stage I and II in CRC; non‐advanced and advanced AD; NA and AD‐CRC, CRC patients with adenoma history) in Disease‐enriched cluster (A), in Disease‐lost cluster (B) and in AD‐lost cluster (C).
**Fig. S5.** KEGG enrichment analysis of significant 5hmC regulated regions increased (left and red) and decreased (right and blue) between CRC and healthy control groups (A), between adenoma and healthy control groups (B), and between CRC and adenoma groups (C).
**Fig. S6.** Volcano plots of all plasma 5hmC DhMRs (A) between CRC and healthy control groups, (B) between adenoma and healthy control groups and (C) between CRC and adenoma groups.
**Fig. S7.** Heatmap of the top 20 DhMRs (A) between tumor and healthy control groups, (B) between adenoma and healthy control groups and (C) between tumor and adenoma groups.

**Table S1.** Clinical characteristics of colorectal cancer and precancerous adenoma patients and healthy controls.

**Table S2.** Annotated genes in 4 clusters, Disease‐enriched, AD‐enriched, Disease‐lost and AD‐lost.

**Table S3.** Annotated genes of differential 5hmC change regions (DhMRs) in the overlaps of 4 clusters.

**Table S4.** Statistics of differential 5hmC change regions (DhMRs) in cfDNAs in three comparisons, CRC vs HC groups, AD vs HC groups, CRC vs AD groups.

**Table S5.** Genes annotated in the top 20 differential hMRs (DhMRs) of cfDNA in three comparison sets, CRC group vs HC group, CRC group vs AD group and AD group vs HC group.

**Table S6.** GO enrichment and annotation of DhMRs of 4 clusters.

Supplementary Material

## Data Availability

All of the raw and processed data used in this study have been uploaded to the Genome Sequence Archive depository [47] with the Accession Number (CRA002181). The R code related to classifier detection and modeling is available upon request.

## References

[1] Bray F , Ferlay J , Soerjomataram I , Siegel RL , Torre LA & Jemal A (2018) Global cancer statistics 2018: GLOBOCAN estimates of incidence and mortality worldwide for 36 cancers in 185 countries. CA Cancer J Clin 68, 394–424. 30207593 10.3322/caac.21492

[2] Siegel RL , Miller KD & Jemal A (2019) Cancer statistics, 2019. CA Cancer J Clin 69, 7–34. 30620402 10.3322/caac.21551

[3] Strum WB (2016) Colorectal adenomas. N Engl J Med 375, 389–390. 10.1056/NEJMc1604867 27464213

[4] Corley DA , Jensen CD , Marks AR , Zhao WK , Lee JK , Doubeni CA , Zauber AG , de Boer J , Fireman BH , Schottinger JE *et al* (2014) Adenoma detection rate and risk of colorectal cancer and death. N Engl J Med 370, 1298–1306. 24693890 10.1056/NEJMoa1309086 PMC4036494

[5] Lieberman D , Moravec M , Holub J , Michaels L & Eisen G (2008) Polyp size and advanced histology in patients undergoing colonoscopy screening: implications for CT colonography. Gastroenterology 135, 1100–1105. 18691580 10.1053/j.gastro.2008.06.083 PMC2581902

[6] Yeh P , Hunter T , Sinha D , Ftouni S , Wallach E , Jiang D , Chan YC , Wong SQ , Silva MJ , Vedururu R *et al* (2017) Circulating tumour DNA reflects treatment response and clonal evolution in chronic lymphocytic leukaemia. Nat Commun 8, 14756. 28303898 10.1038/ncomms14756 PMC5357854

[7] Weisenberger DJ , Liang G & Lenz HJ (2018) DNA methylation aberrancies delineate clinically distinct subsets of colorectal cancer and provide novel targets for epigenetic therapies. Oncogene 37, 566–577. 28991233 10.1038/onc.2017.374 PMC7491233

[8] Cohen JD , Li L , Wang Y , Thoburn C , Afsari B , Danilova L , Douville C , Javed AA , Wong F , Mattox A *et al* (2018) Detection and localization of surgically resectable cancers with a multi‐analyte blood test. Science 359, 926–930. 29348365 10.1126/science.aar3247 PMC6080308

[9] Flavahan WA , Gaskell E & Bernstein BE (2017) Epigenetic plasticity and the hallmarks of cancer. Science 357, eaal2380. 28729483 10.1126/science.aal2380 PMC5940341

[10] Hao X , Luo H , Krawczyk M , Wei W , Wang W , Wang J , Flagg K , Hou J , Zhang H , Yi S *et al* (2017) DNA methylation markers for diagnosis and prognosis of common cancers. Proc Natl Acad Sci USA 114, 7414–7419. 28652331 10.1073/pnas.1703577114 PMC5514741

[11] Xu RH , Wei W , Krawczyk M , Wang W , Luo H , Flagg K , Yi S , Shi W , Quan Q , Li K *et al* (2017) Circulating tumour DNA methylation markers for diagnosis and prognosis of hepatocellular carcinoma. Nat Mater 16, 1155–1161. 29035356 10.1038/nmat4997

[12] Mooijman D , Dey SS , Boisset JC , Crosetto N & van Oudenaarden A (2016) Single‐cell 5hmC sequencing reveals chromosome‐wide cell‐to‐cell variability and enables lineage reconstruction. Nat Biotechnol 34, 852–856. 27347753 10.1038/nbt.3598

[13] Song CX , Szulwach KE , Fu Y , Dai Q , Yi C , Li X , Li Y , Chen CH , Zhang W , Jian X *et al* (2011) Selective chemical labeling reveals the genome‐wide distribution of 5‐hydroxymethylcytosine. Nat Biotechnol 29, 68–72. 21151123 10.1038/nbt.1732 PMC3107705

[14] Han D , Lu X , Shih AH , Nie J , You Q , Xu MM , Melnick AM , Levine RL & He C (2016) A highly sensitive and robust method for genome‐wide 5hmC profiling of rare cell populations. Mol Cell 63, 711–719. 27477909 10.1016/j.molcel.2016.06.028 PMC4992443

[15] Dong C , Chen J , Zheng J , Liang Y , Yu T , Liu Y , Gao F , Long J , Chen H , Zhu Q *et al* (2020) 5‐Hydroxymethylcytosine signatures in circulating cell‐free DNA as diagnostic and predictive biomarkers for coronary artery disease. Clin Epigenetics 12, 17. 31964422 10.1186/s13148-020-0810-2 PMC6974971

[16] Tian X , Sun B , Chen C , Gao C , Zhang J , Lu X , Wang L , Li X , Xing Y , Liu R *et al* (2018) Circulating tumor DNA 5‐hydroxymethylcytosine as a novel diagnostic biomarker for esophageal cancer. Cell Res 28, 597–600. 29467383 10.1038/s41422-018-0014-x PMC5951904

[17] Zhang J , Han X , Gao C , Xing Y , Qi Z , Liu R , Wang Y , Zhang X , Yang YG , Li X *et al* (2018) 5‐Hydroxymethylome in circulating cell‐free DNA as a potential biomarker for non‐small‐cell lung cancer. Genomics Proteomics Bioinformatics 16, 187–199. 30010036 10.1016/j.gpb.2018.06.002 PMC6076378

[18] Li W , Zhang X , Lu X , You L , Song Y , Luo Z , Zhang J , Nie J , Zheng W , Xu D *et al* (2017) 5‐Hydroxymethylcytosine signatures in circulating cell‐free DNA as diagnostic biomarkers for human cancers. Cell Res 27, 1243–1257. 28925386 10.1038/cr.2017.121 PMC5630683

[19] Gao P , Lin S , Cai M , Zhu Y , Song Y , Sui Y , Lin J , Liu J , Lu X , Zhong Y *et al* (2019) 5‐Hydroxymethylcytosine profiling from genomic and cell‐free DNA for colorectal cancers patients. J Cell Mol Med 23, 3530–3537. 30912288 10.1111/jcmm.14252 PMC6484304

[20] Grytten I , Rand KD , Nederbragt AJ , Storvik GO , Glad IK & Sandve GK (2019) Graph peak caller: calling ChIP‐seq peaks on graph‐based reference genomes. PLoS Comput Biol 15, e1006731. 30779737 10.1371/journal.pcbi.1006731 PMC6396939

[21] Heinz S , Benner C , Spann N , Bertolino E , Lin YC , Laslo P , Cheng JX , Murre C , Singh H & Glass CK (2010) Simple combinations of lineage‐determining transcription factors prime cis‐regulatory elements required for macrophage and B cell identities. Mol Cell 38, 576–589. 20513432 10.1016/j.molcel.2010.05.004 PMC2898526

[22] Cavalcante RG & Sartor MA (2017) annotatr: genomic regions in context. Bioinformatics 33, 2381–2383. 28369316 10.1093/bioinformatics/btx183 PMC5860117

[23] Shen L , Shao N , Liu X & Nestler E (2014) ngs.plot: quick mining and visualization of next‐generation sequencing data by integrating genomic databases. BMC Genom 15, 284. 10.1186/1471-2164-15-284 PMC4028082 24735413

[24] Robinson MD , McCarthy DJ & Smyth GK (2010) edgeR: a Bioconductor package for differential expression analysis of digital gene expression data. Bioinformatics 26, 139–140. 19910308 10.1093/bioinformatics/btp616 PMC2796818

[25] Galili T , O'Callaghan A , Sidi J & Sievert C (2018) heatmaply: an R package for creating interactive cluster heatmaps for online publishing. Bioinformatics 34, 1600–1602. 29069305 10.1093/bioinformatics/btx657 PMC5925766

[26] Song CX , Yin S , Ma L , Wheeler A , Chen Y , Zhang Y , Liu B , Xiong J , Zhang W , Hu J *et al* (2017) 5‐Hydroxymethylcytosine signatures in cell‐free DNA provide information about tumor types and stages. Cell Res 27, 1231–1242. 28820176 10.1038/cr.2017.106 PMC5630676

[27] Mellen M , Ayata P , Dewell S , Kriaucionis S & Heintz N (2012) MeCP2 binds to 5hmC enriched within active genes and accessible chromatin in the nervous system. Cell 151, 1417–1430. 23260135 10.1016/j.cell.2012.11.022 PMC3653293

[28] Li Y , Liu X , Tang H , Yang H & Meng X (2017) RNA sequencing uncovers molecular mechanisms underlying pathological complete response to chemotherapy in patients with operable breast cancer. Med Sci Monit 23, 4321–4327. 28880852 10.12659/MSM.903272 PMC5600194

[29] Vigneswaran N & Williams MD (2014) Epidemiologic trends in head and neck cancer and aids in diagnosis. Oral Maxillofac Surg Clin North Am 26, 123–141. 24794262 10.1016/j.coms.2014.01.001 PMC4040236

[30] Printz C (2014) Stronger nicotine addiction associated with higher risk of lung cancer. Cancer 120, 3591. 10.1002/cncr.29127 25410487

[31] Li H , Liu JW , Liu S , Yuan Y & Sun LP (2017) Bioinformatics‐based identification of methylated‐differentially expressed genes and related pathways in gastric cancer. Dig Dis Sci 62, 3029–3039. 28914394 10.1007/s10620-017-4740-6

[32] Zeng JH , Xiong DD , Pang YY , Zhang Y , Tang RX , Luo DZ & Chen G (2017) Identification of molecular targets for esophageal carcinoma diagnosis using miRNA‐seq and RNA‐seq data from The Cancer Genome Atlas: a study of 187 cases. Oncotarget 8, 35681–35699. 28415685 10.18632/oncotarget.16051 PMC5482608

[33] Biankin AV , Waddell N , Kassahn KS , Gingras MC , Muthuswamy LB , Johns AL , Miller DK , Wilson PJ , Patch AM , Wu J *et al* (2012) Pancreatic cancer genomes reveal aberrations in axon guidance pathway genes. Nature 491, 399–405. 23103869 10.1038/nature11547 PMC3530898

[34] Kellermeyer R , Heydman LM , Mastick GS & Kidd T (2018) The role of apoptotic signaling in axon guidance. J Dev Biol 6 10.3390/jdb6040024 PMC6316149 30340315

[35] Polenghi A , Nieus T , Guazzi S , Gorostiza P , Petrini EM & Barberis A (2020) Kainate receptor activation shapes short‐term synaptic plasticity by controlling receptor lateral mobility at glutamatergic synapses. Cell Rep 31, 107735. 32521260 10.1016/j.celrep.2020.107735 PMC7296349

[36] Shen Q , Wang H & Zhang L (2018) Effect of exogenous p51a gene on the growth and chemo sensitivity of human lung adenocarcinoma cell lines. Artif Cells Nanomed Biotechnol 46, S383–S388. 30095026 10.1080/21691401.2018.1494600

[37] Yang F , Aubele M , Walch A , Gross E , Napieralski R , Zhao S , Ahmed N , Kiechle M , Reuning U , Dorn J *et al* (2017) Tissue kallikrein‐related peptidase 4 (KLK4), a novel biomarker in triple‐negative breast cancer. Biol Chem 398, 1151–1164. 28755528 10.1515/hsz-2017-0122

[38] Cui Z , Cui Y , Luo G , Yang S , Ling X , Lou Y & Sun X (2017) Kallikrein‐related peptidase 4 contributes to the tumor metastasis of oral squamous cell carcinoma. Biosci Biotechnol Biochem 81, 1768–1777. 28743213 10.1080/09168451.2017.1356216

[39] Kryza T , Silva LM , Bock N , Fuhrman‐Luck RA , Stephens CR , Gao J , Samaratunga H , Australian Prostate Cancer, B. , Lawrence MG , Hooper JD *et al* (2017) Kallikrein‐related peptidase 4 induces cancer‐associated fibroblast features in prostate‐derived stromal cells. Mol Oncol 11, 1307–1329. 28510269 10.1002/1878-0261.12075 PMC5623815

[40] Kontos CK , Chantzis D , Papadopoulos IN & Scorilas A (2013) Kallikrein‐related peptidase 4 (KLK4) mRNA predicts short‐term relapse in colorectal adenocarcinoma patients. Cancer Lett 330, 106–112. 23201139 10.1016/j.canlet.2012.11.036

[41] Uribe‐Lewis S , Stark R , Carroll T , Dunning MJ , Bachman M , Ito Y , Stojic L , Halim S , Vowler SL , Lynch AG *et al* (2015) 5‐hydroxymethylcytosine marks promoters in colon that resist DNA hypermethylation in cancer. Genome Biol 16, 69. 25853800 10.1186/s13059-015-0605-5 PMC4380107

[42] Zhang LT , Zhang LJ , Zhang JJ , Ye XX , Xie AM , Chen LY , Kang JX & Cai C (2013) Quantification of the sixth DNA base 5‐hydroxymethylcytosine in colorectal cancer tissue and C‐26 cell line. Bioanalysis 5, 839–845. 23534428 10.4155/bio.13.28

[43] Lee‐Six H , Olafsson S , Ellis P , Osborne RJ , Sanders MA , Moore L , Georgakopoulos N , Torrente F , Noorani A , Goddard M *et al* (2019) The landscape of somatic mutation in normal colorectal epithelial cells. Nature 574, 532–537. 31645730 10.1038/s41586-019-1672-7

[44] Kim TM , An CH , Rhee JK , Jung SH , Lee SH , Baek IP , Kim MS , Lee SH & Chung YJ (2015) Clonal origins and parallel evolution of regionally synchronous colorectal adenoma and carcinoma. Oncotarget 6, 27725–27735. 26336987 10.18632/oncotarget.4834 PMC4695021

[45] Kreso A , O'Brien CA , van Galen P , Gan OI , Notta F , Brown AM , Ng K , Ma J , Wienholds E , Dunant C *et al* (2013) Variable clonal repopulation dynamics influence chemotherapy response in colorectal cancer. Science 339, 543–548. 23239622 10.1126/science.1227670 PMC9747244

[46] Lamprecht S , Schmidt EM , Blaj C , Hermeking H , Jung A , Kirchner T & Horst D (2017) Multicolor lineage tracing reveals clonal architecture and dynamics in colon cancer. Nat Commun 8, 1406. 29127276 10.1038/s41467-017-00976-9 PMC5681634

[47] Wang Y , Song F , Zhu J , Zhang S , Yang Y , Chen T , Tang B , Dong L , Ding N , Zhang Q *et al* (2017) GSA: Genome sequence archive. Genomics Proteomics Bioinformatics 15, 14–18. 28387199 10.1016/j.gpb.2017.01.001 PMC5339404

