# Supplementary material for: 5‐Hydroxymethylcytosine signature in circulating cell‐free DNA as a potential diagnostic factor for early‐stage colorectal cancer and precancerous adenoma
Source: Mol Oncol. 2020 Nov 14;15(1):138–50. doi: 10.1002/1878-0261.12833 (PMC7782095; doi:10.1002/1878-0261.12833)
Supplement: Supplementary file 1 — Fig. S1. Representative images of colorectal cancer (Stage I and Stage II), advanced tubular adenoma, Villus tubular adenoma and non‐advanced adenoma tissue. Fig. S2. General procedure of 5hmC sequencing profiling from gDNA and cfDNA. Fig. S3. Kruskal‐Wallis H‐test among CRC, AD and HC groups (A) for age distributions, (B) for gender distributions. Fig. S4. Heatmap of differentially 5hmC hMRs (FC ≥ 2 and p ≤ 0.01) with stage status (Stage I and II in CRC; non‐advanced and advanced AD; NA and AD‐CRC, CRC patients with adenoma history) in Disease‐enriched cluster (A), in Disease‐lost cluster (B) and in AD‐lost cluster (C). Fig. S5. KEGG enrichment analysis of significant 5hmC regulated regions increased (left and red) and decreased (right and blue) between CRC and healthy control groups (A), between adenoma and healthy control groups (B), and between CRC and adenoma groups (C). Fig. S6. Volcano plots of all plasma 5hmC DhMRs (A) between CRC and healthy control groups, (B) between adenoma and healthy control groups and (C) between CRC and adenoma groups. Fig. S7. Heatmap of the top 20 DhMRs (A) between tumor and healthy control groups, (B) between adenoma and healthy control groups and (C) between tumor and adenoma groups. [file MOL2-15-138-s001.pptx]

## Slide 1
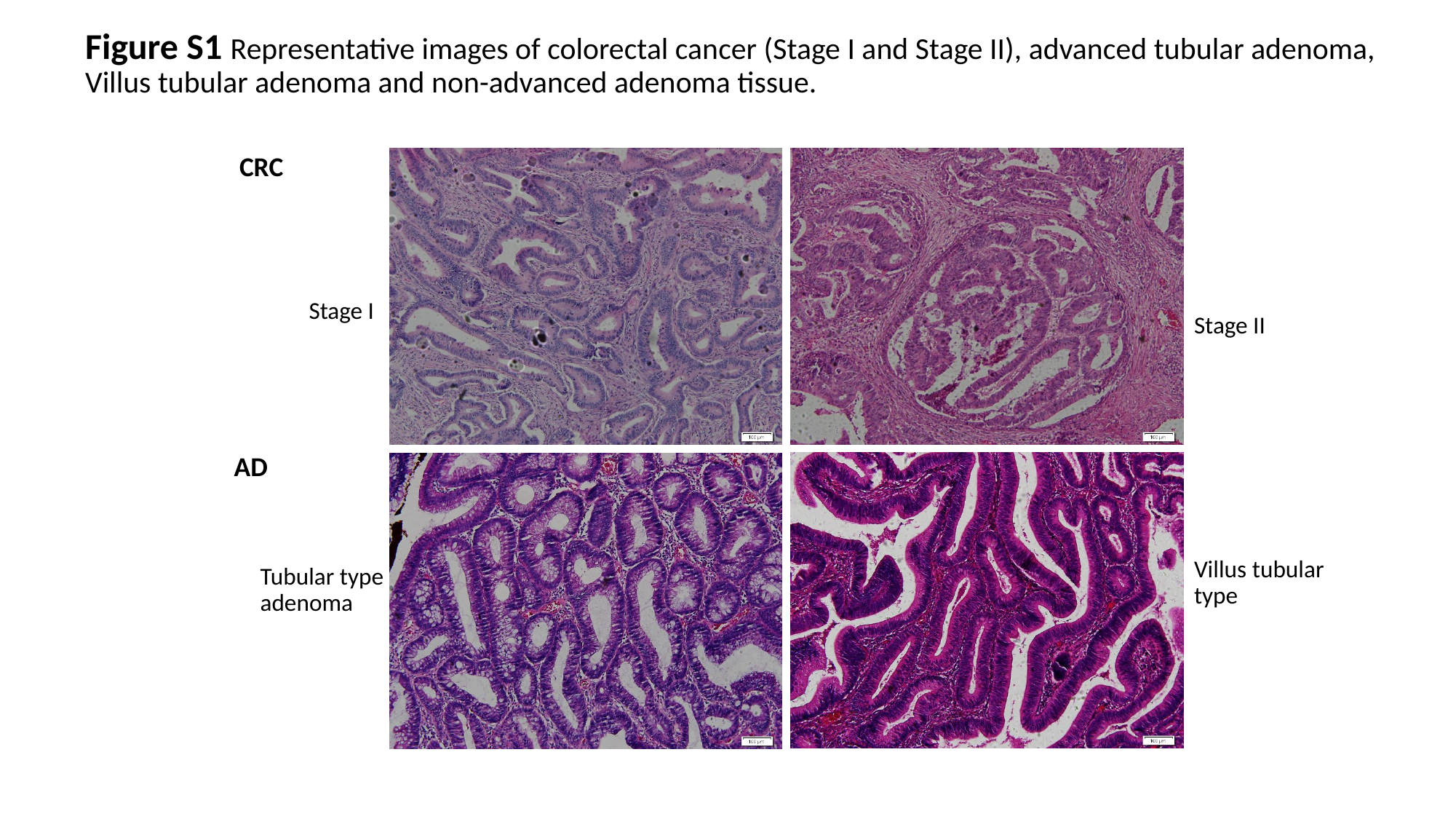

# Figure S1 Representative images of colorectal cancer (Stage I and Stage II), advanced tubular adenoma, Villus tubular adenoma and non-advanced adenoma tissue.
CRC
Stage I
Stage II
AD
Villus tubular type
Tubular type adenoma

## Slide 2
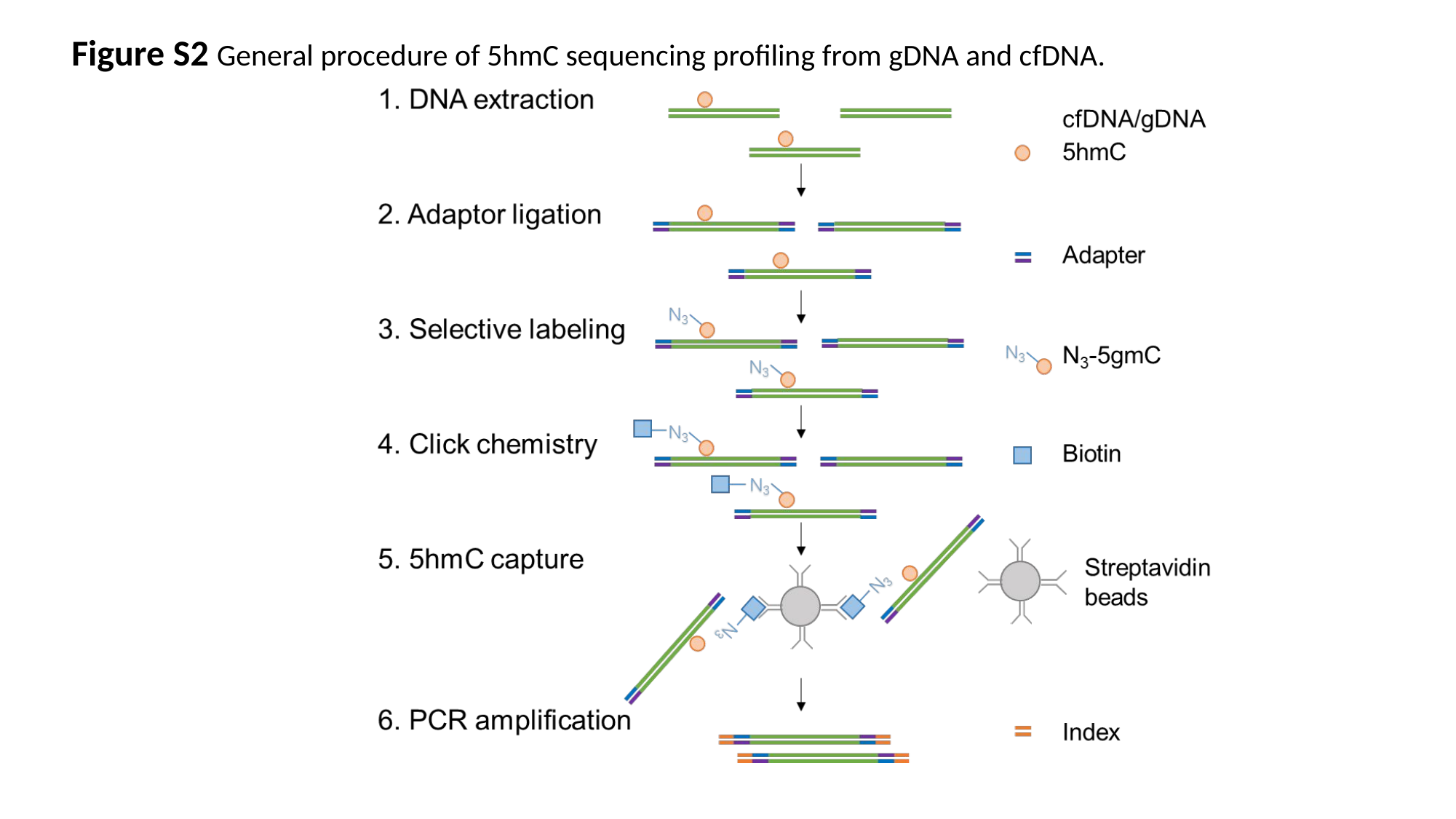

Figure S2 General procedure of 5hmC sequencing profiling from gDNA and cfDNA.

## Slide 3
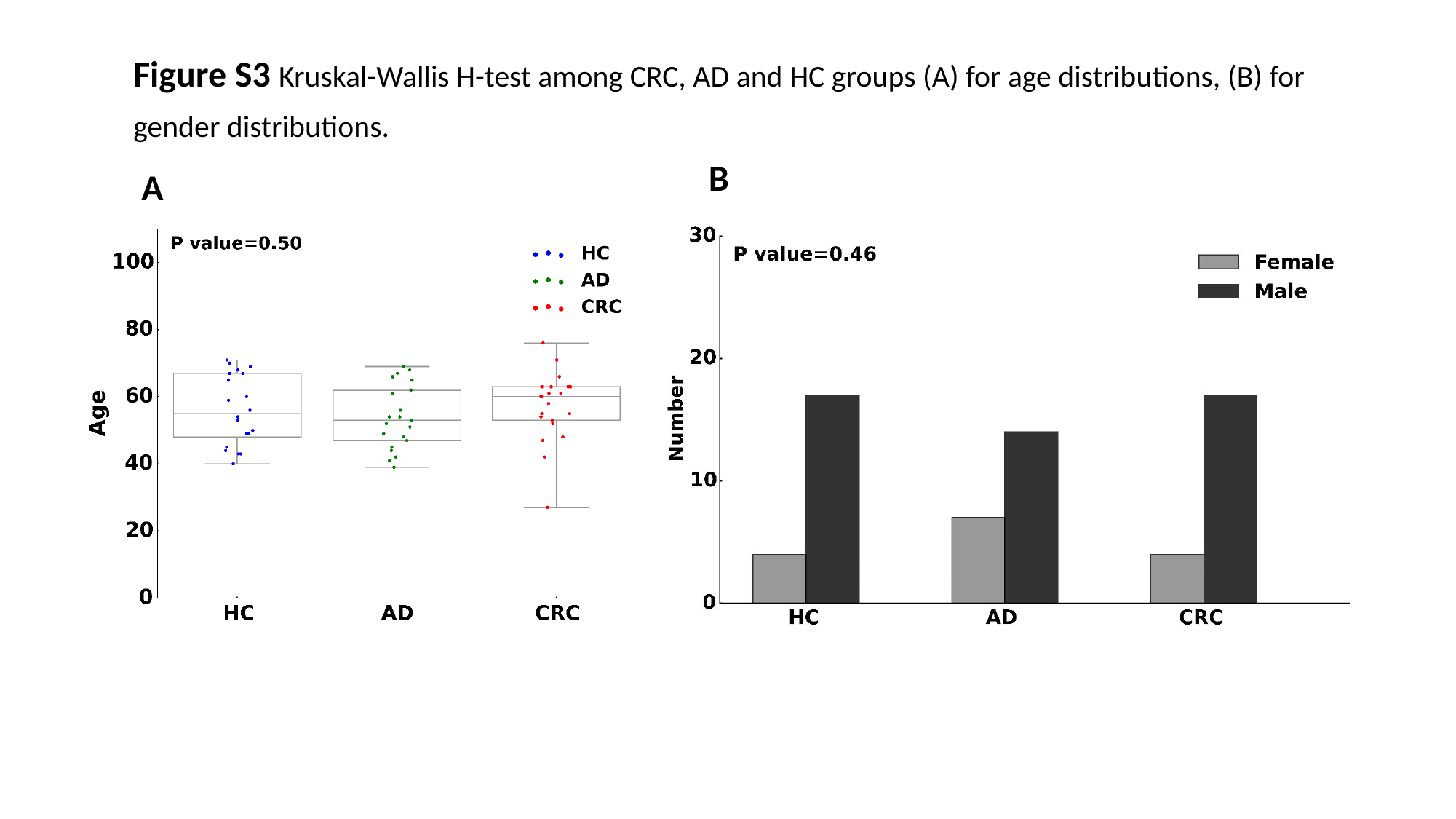

Figure S3 Kruskal-Wallis H-test among CRC, AD and HC groups (A) for age distributions, (B) for gender distributions.
B
A

## Slide 4
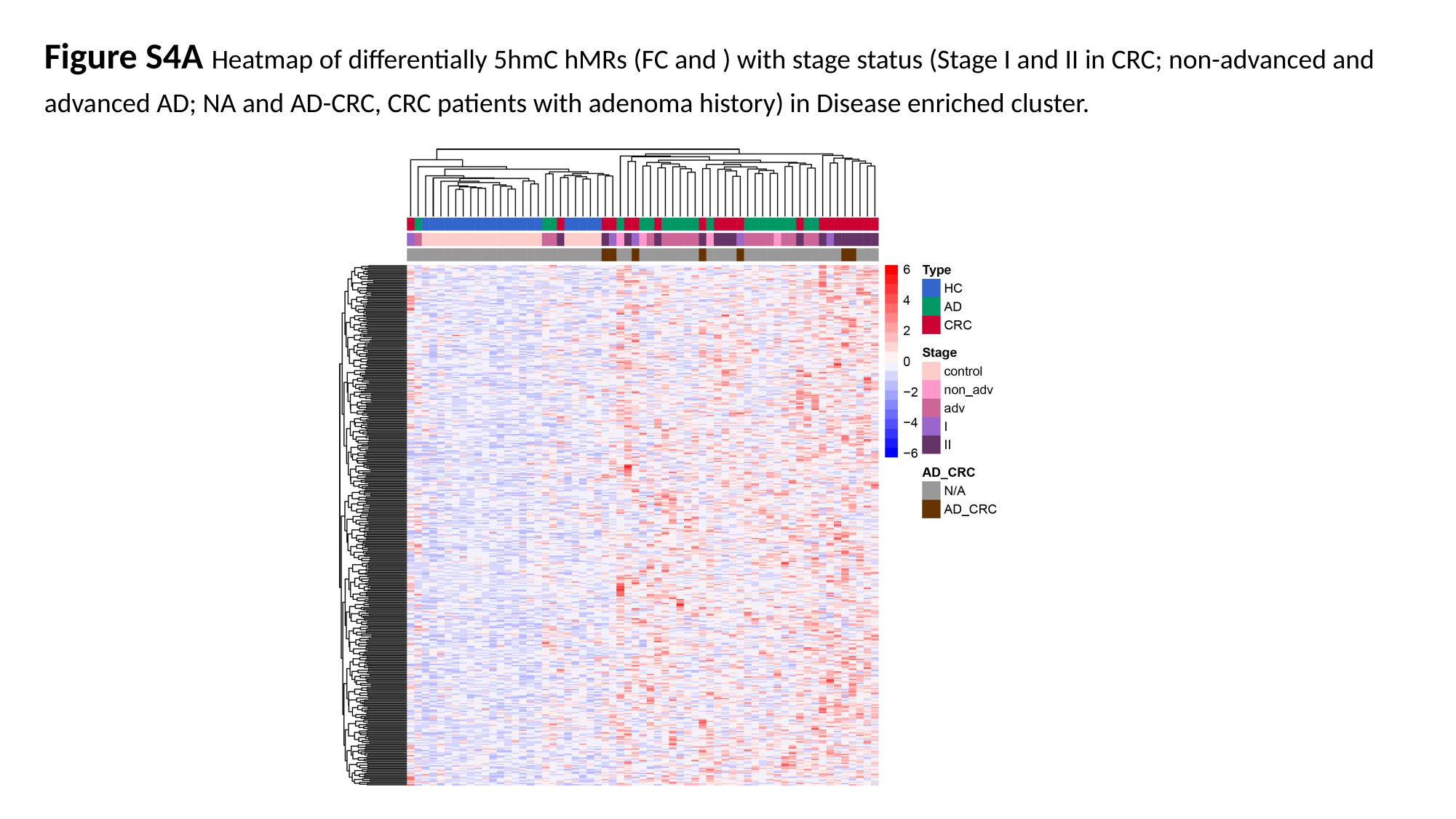

## Slide 5
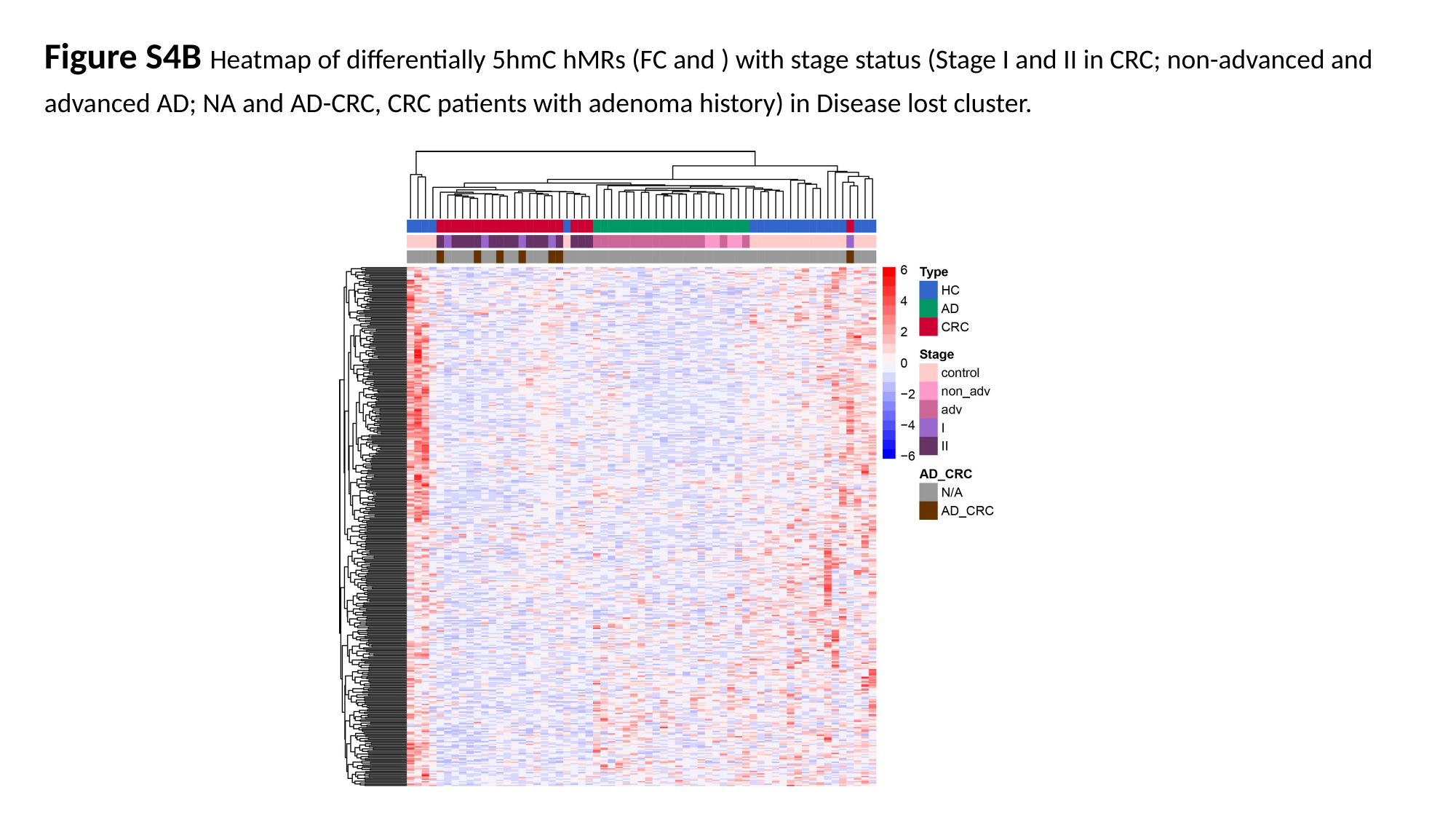

## Slide 6
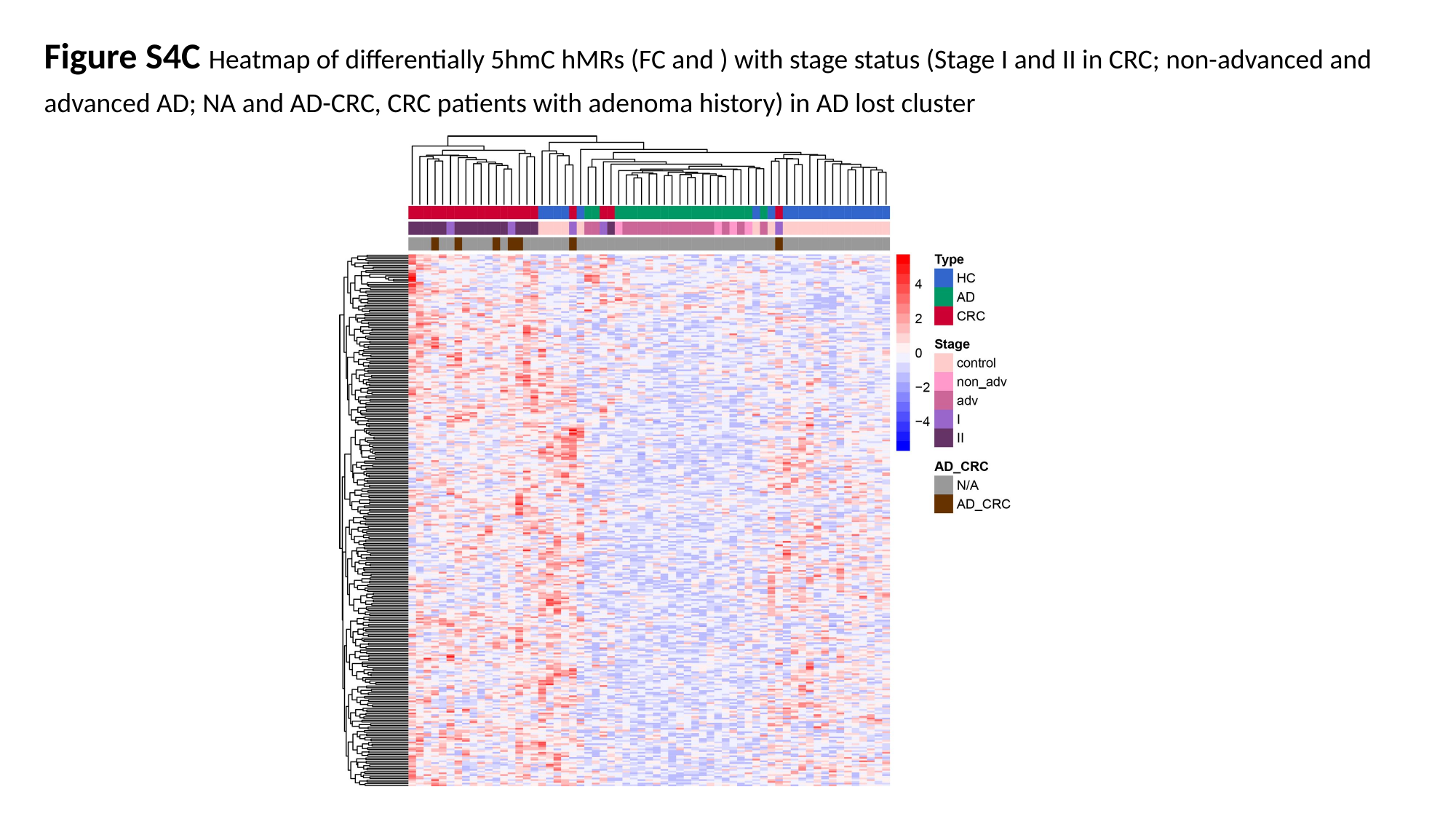

## Slide 7
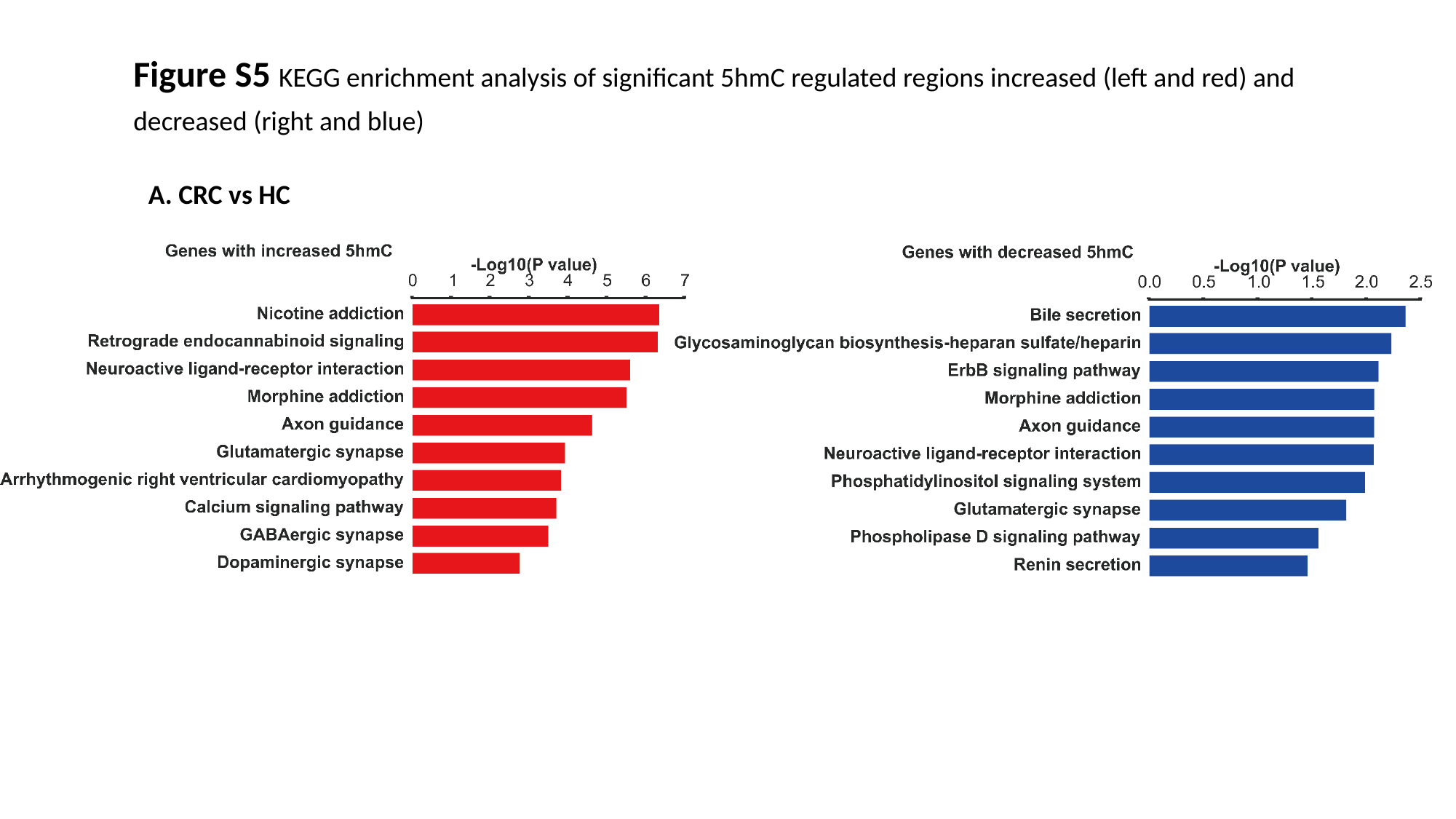

Figure S5 KEGG enrichment analysis of significant 5hmC regulated regions increased (left and red) and decreased (right and blue)
A. CRC vs HC

## Slide 8
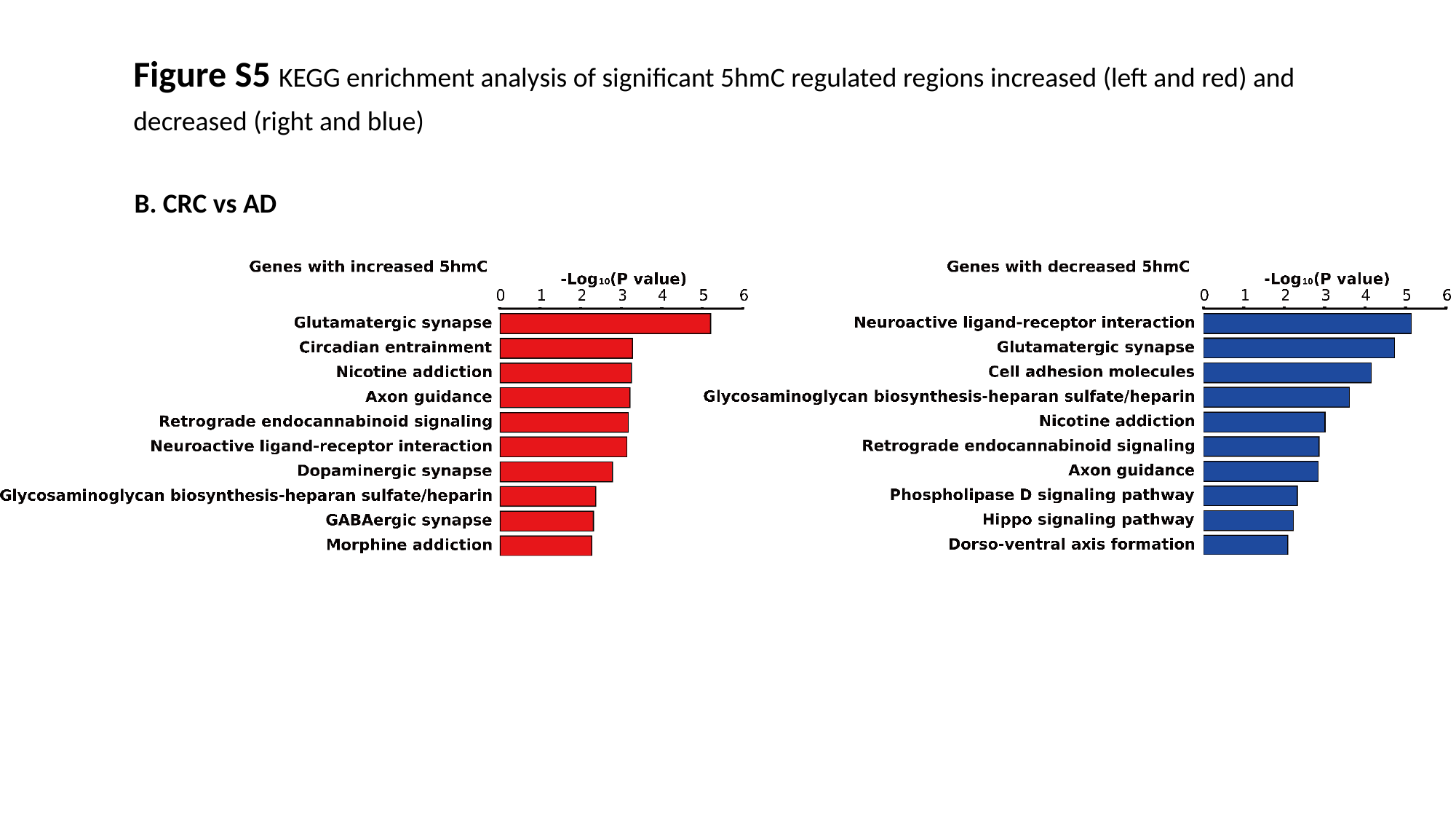

Figure S5 KEGG enrichment analysis of significant 5hmC regulated regions increased (left and red) and decreased (right and blue)
B. CRC vs AD

## Slide 9
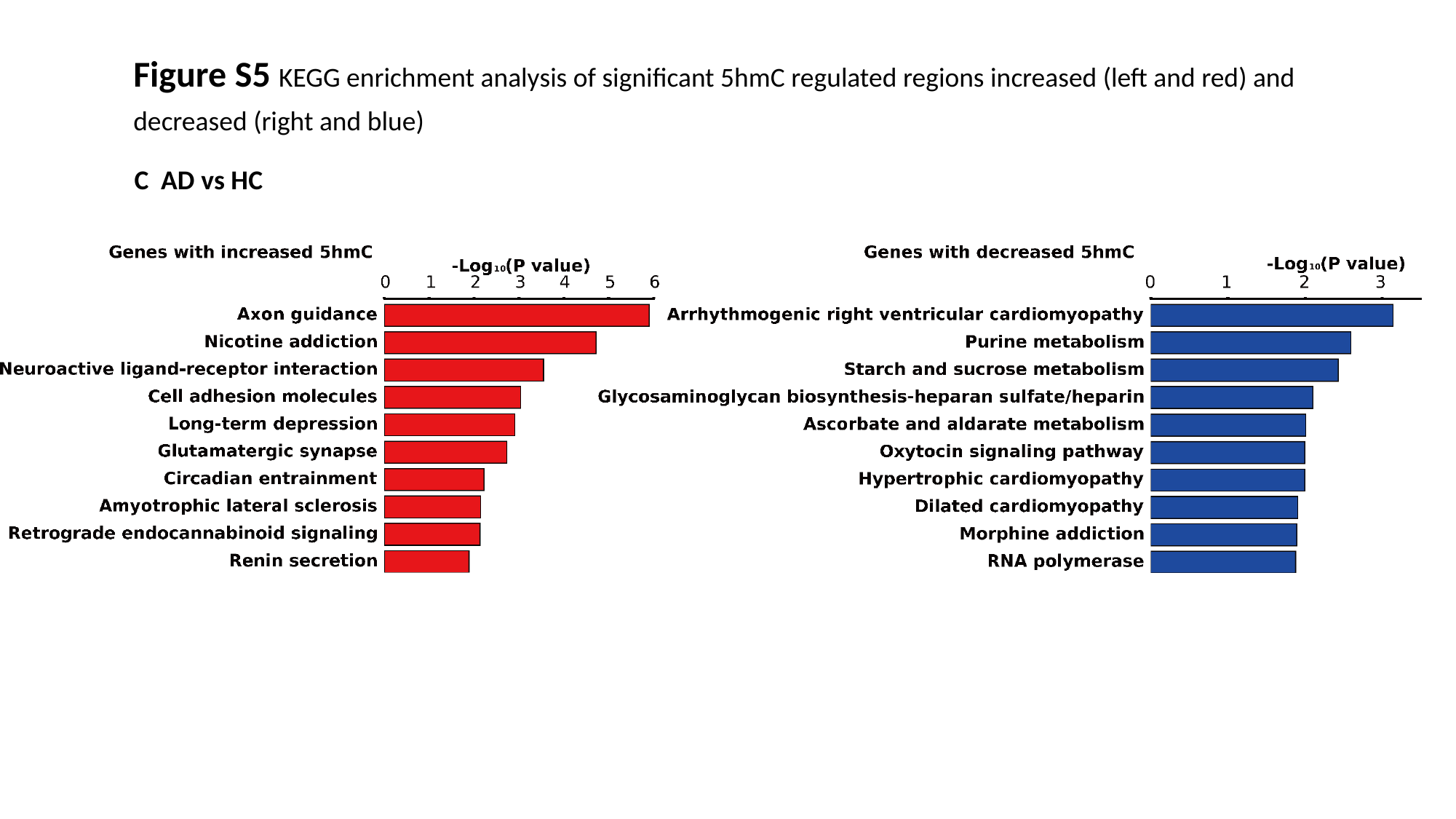

Figure S5 KEGG enrichment analysis of significant 5hmC regulated regions increased (left and red) and decreased (right and blue)
C AD vs HC

## Slide 10
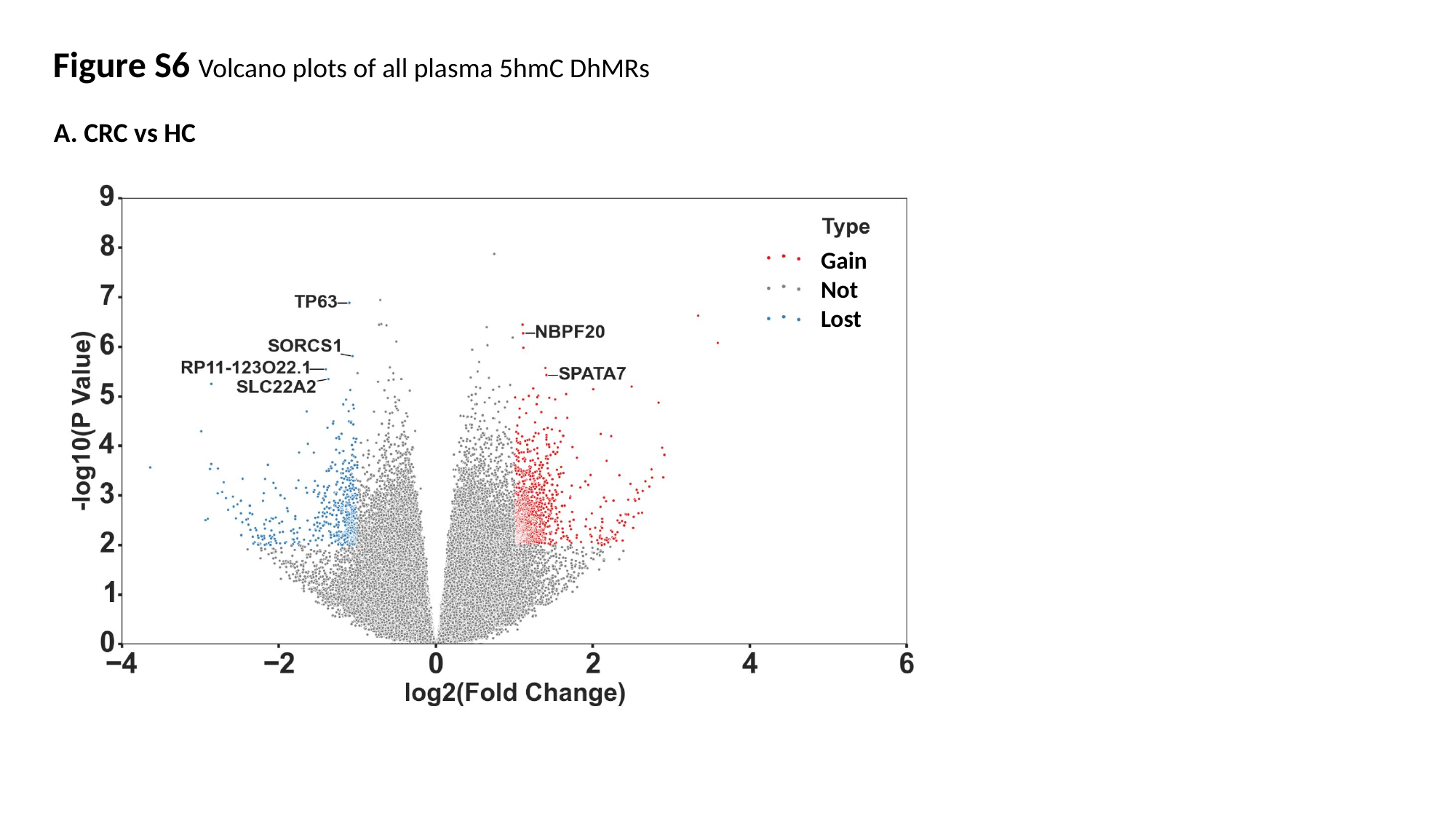

Figure S6 Volcano plots of all plasma 5hmC DhMRs
A. CRC vs HC
Gain
Not
Lost

## Slide 11
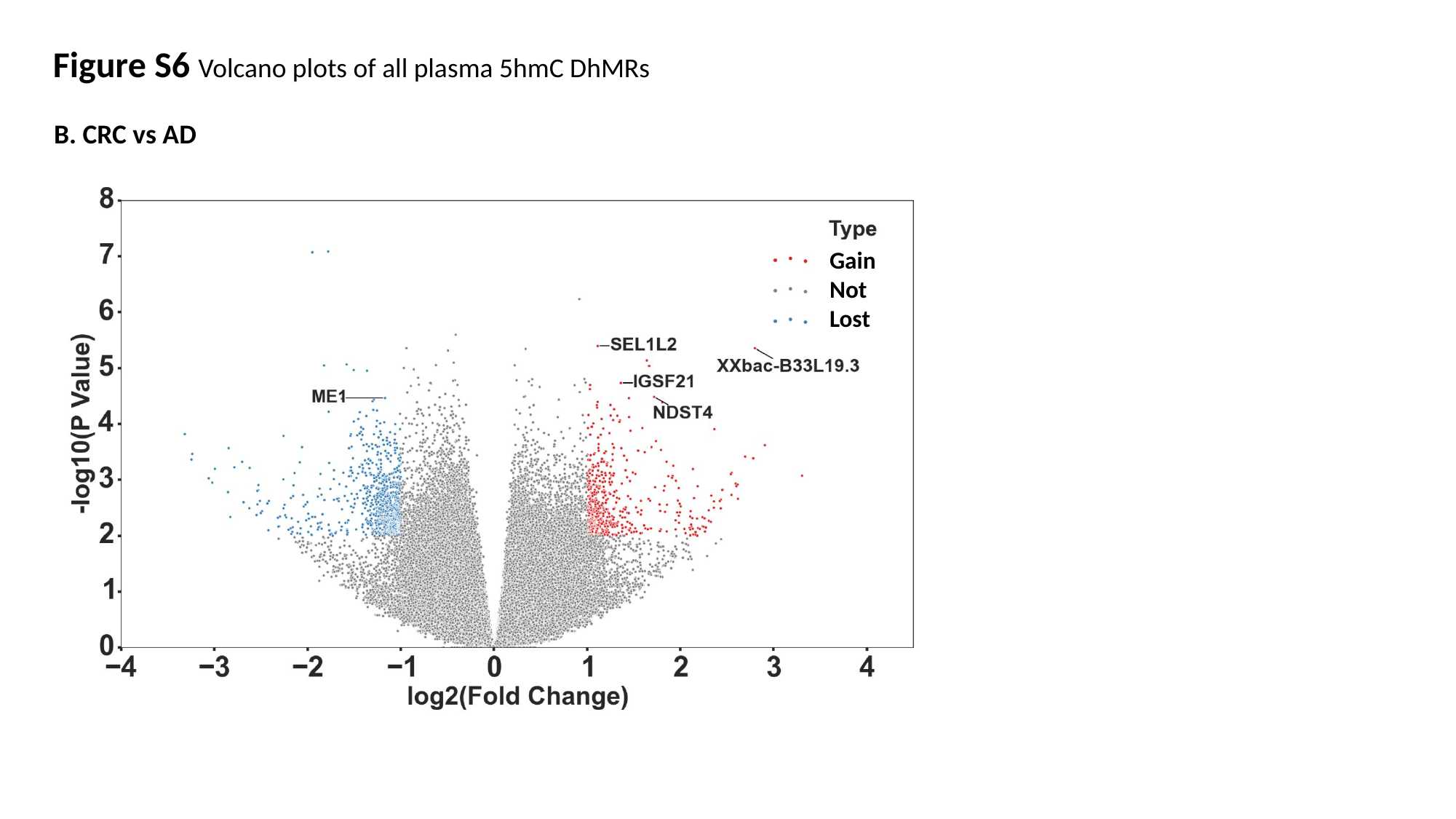

Figure S6 Volcano plots of all plasma 5hmC DhMRs
B. CRC vs AD
Gain
Not
Lost

## Slide 12
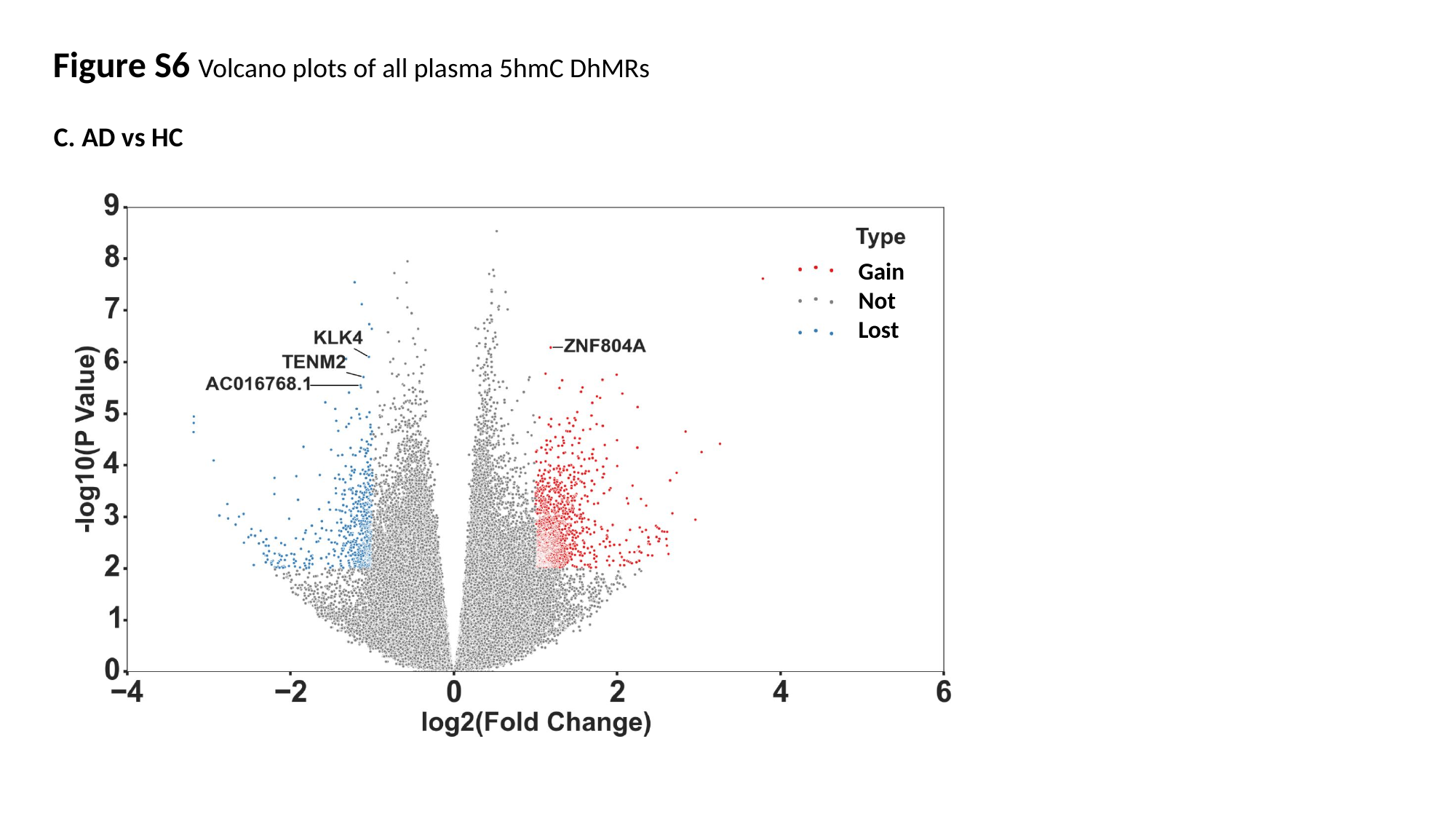

Figure S6 Volcano plots of all plasma 5hmC DhMRs
C. AD vs HC
Gain
Not
Lost

## Slide 13
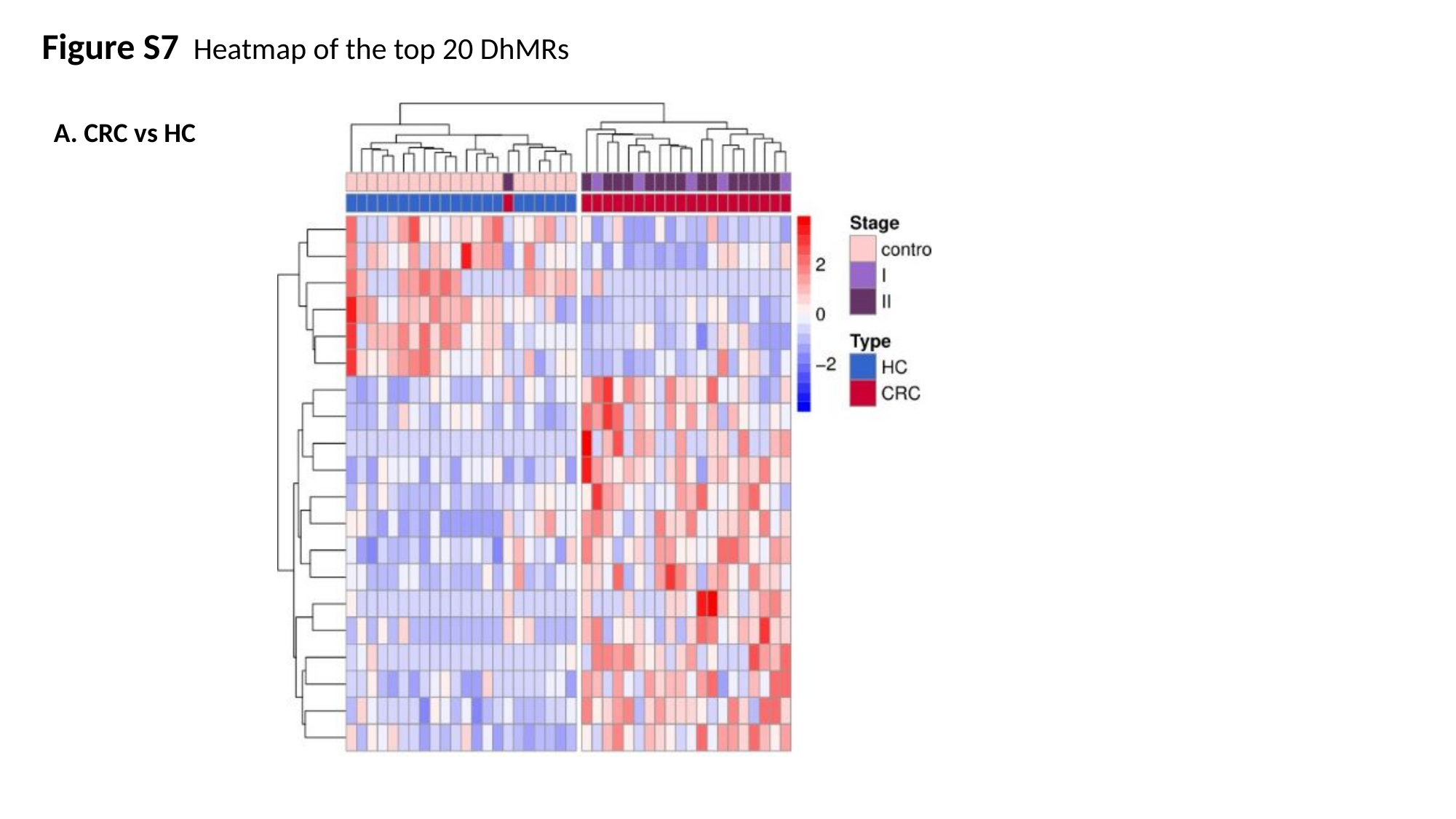

Figure S7 Heatmap of the top 20 DhMRs
A. CRC vs HC

## Slide 14
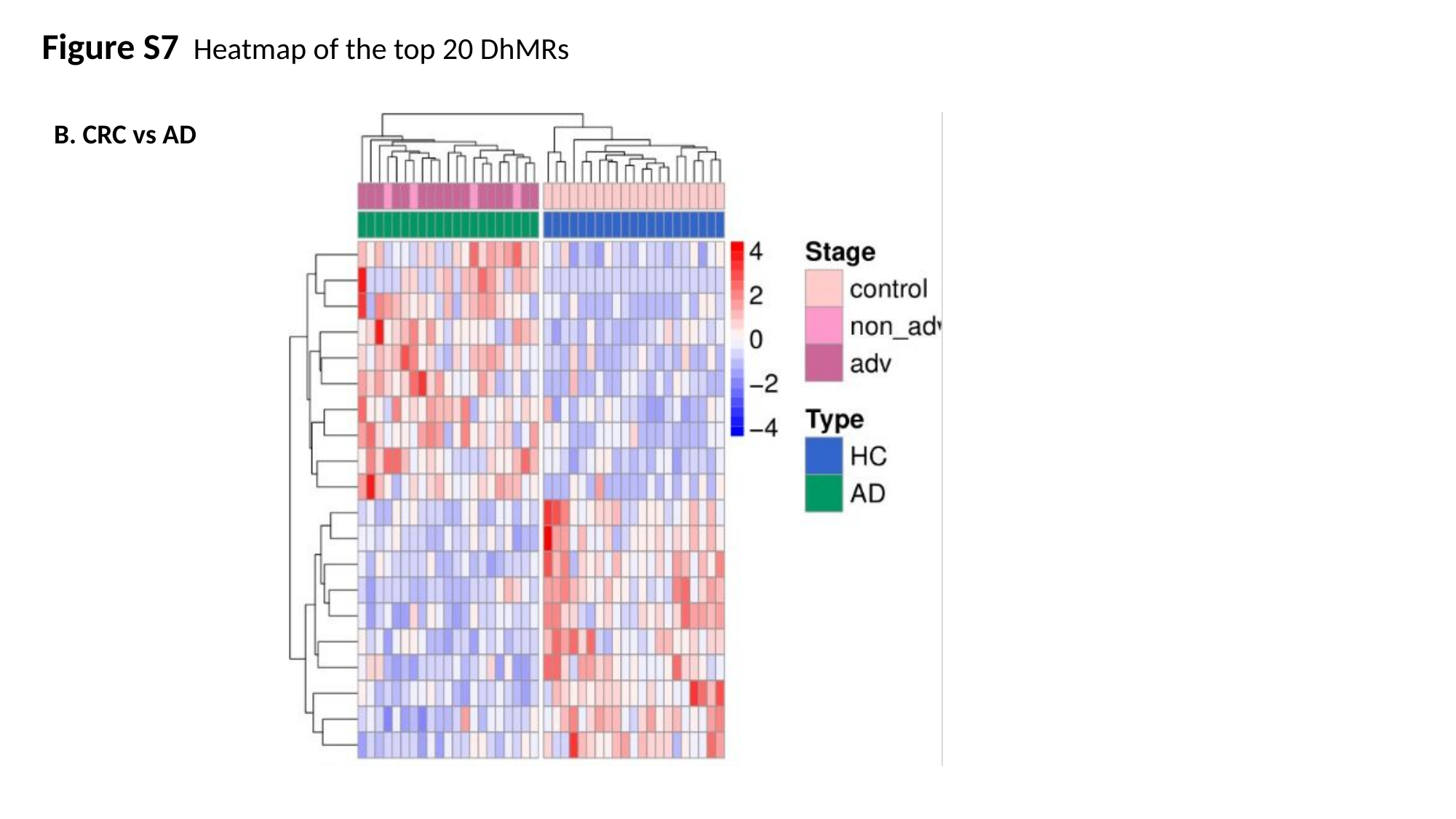

Figure S7 Heatmap of the top 20 DhMRs
B. CRC vs AD

## Slide 15
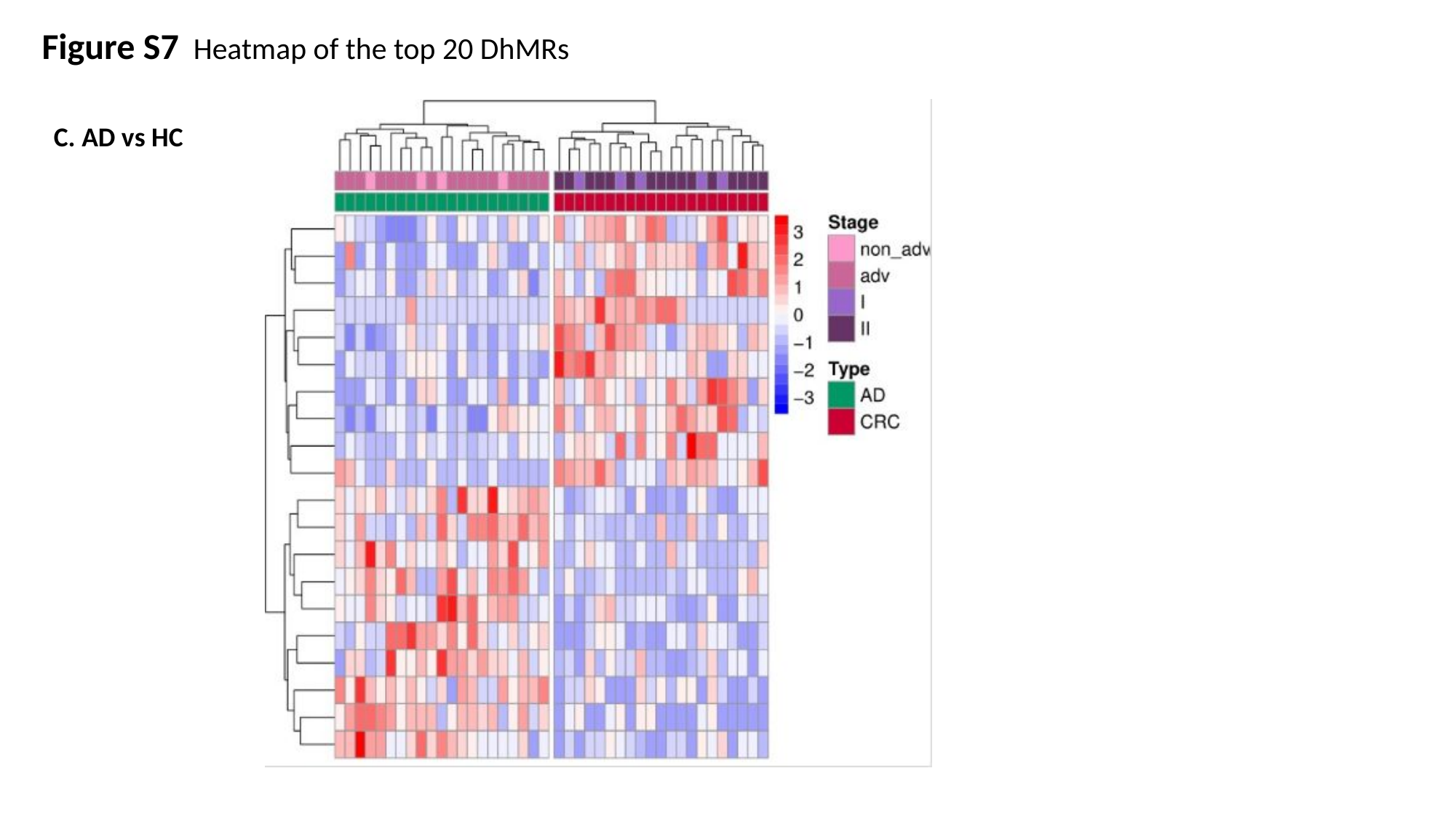

Figure S7 Heatmap of the top 20 DhMRs
C. AD vs HC
