# Supplementary material for: 5‐Hydroxymethylcytosine signature in circulating cell‐free DNA as a potential diagnostic factor for early‐stage colorectal cancer and precancerous adenoma
Source: Mol Oncol. 2020 Nov 14;15(1):138–50. doi: 10.1002/1878-0261.12833 (PMC7782095; doi:10.1002/1878-0261.12833)
Supplement: Supplementary file 8 — Supplementary Material [file MOL2-15-138-s008.docx]

**Supplementary Information**

**Table S1**. Clinical characteristics of colorectal cancer and precancerous adenoma patients and healthy controls.

**Table S2**. Genes annotated in 4 clusters, Disease enriched, AD enriched, Disease lost and AD lost.

**Table S3**. Annotated genes of differential 5hmC change regions DhMRs in the overlaps of 4 clusters.

**Table S4**. Statistics of DhMRs among three comparison sets.

**Table S5**. Genes annotated in the top 20 differential hMRs (DhMRs) of cfDNA in three comparison sets.

**Table S6**. GO enrichment and annotation of DhMRs of 4 clusters.

**Figure S1**. Representative images of colorectal cancer (Stage I and Stage II), advanced tubular adenoma, Villus tubular adenoma and non-advanced adenoma tissue.

**Figure S2**. General procedure of 5hmC sequencing profiling from gDNA and cfDNA.

**Figure S3**. Kruskal-Wallis H-test among CRC, AD and HC groups (A) for age distributions, (B) for gender distributions.

**Figure S4**. Heatmap of differentially 5hmC hMRs (FC$\geq2$ and $p\leq0.01$) with stage status (Stage I and II in CRC; non-advanced and advanced AD; NA and AD-CRC, CRC patients with adenoma history) in Disease enriched cluster (A), in Disease lost cluster (B) and in AD lost cluster (C). Unsupervised hierarchical clustering was performed across samples and hMRs.

**Figure S5**. KEGG enrichment analysis of significant 5hmC regulated regions increased (left and red) and decreased (right and blue) between CRC and healthy control groups (A), between adenoma and healthy control groups (B), and between CRC and adenoma groups (C).

**Figure S6**. Volcano plots of all plasma 5hmC DhMRs (A) between CRC and healthy control groups, (B) between adenoma and healthy control groups and (C) between CRC and adenoma groups. Red represents 5hmC signal gain or increase, blue represents 5hmC signal lost or decrease. The identified genes in the top 20 hits are shown with their names in each category. TP63, tumor protein 63; NBPF, neuroblastoma breakpoint family member; SORCS1, sortilin related VPS10 domain containing receptor 1; SPATA7, spermatogenesis associated 7; RP11-123O22.1, lncRNA; SLC22A2, solute carrier family 22 member 2; SEL1L2, SEL1L2 adaptor subunit of ERAD E3 ligase; XXbac-B33L19.3, GUSB pseudogene 4; IGSF21, immunoglobin superfamily member 21; NDST4, N-deacetylase/N-sulfotransferase (heparan glucosaminyl) 4; ME1, malic enzyme 1; ZNF804A, zinc finger protein 804A; KLK4, kallikrein related peptidase 4; TENM2, teneurin transmembrane protein 2; AC016768.1, BAC clone RP11-560C7 from 2. HC, healthy control. AD, adenoma. CRC colorectal cancer. Gain, 5hmc-signal increased significantly. NOT, 5hmC-signal without significantly changed. Lost, 5hmC-signal decreased significantly.

**Figure S7**. Heatmap of the top 20 DhMRs (A) between tumor and healthy control groups, (B) between adenoma and healthy control groups and (C) between tumor and adenoma groups. Unsupervised hierarchical clustering was performed across samples and hMRs (FC≥2 and $p$ p≤0.01). HC, healthy control. AD, adenoma. CRC colorectal cancer. Control, healthy control. non-adv, non-advanced. adv, advanced.
